# Estrogen and G protein-coupled estrogen receptor accelerate the progression of benign prostatic hyperplasia by inducing prostatic fibrosis

**DOI:** 10.1038/s41419-022-04979-3

**Published:** 2022-06-07

**Authors:** Yang Yang, Jindong Sheng, Shuai Hu, Yun Cui, Jing Xiao, Wei Yu, Jing Peng, Wenke Han, Qun He, Yu Fan, Yuanjie Niu, Jun Lin, Ye Tian, Chawnshang Chang, Shuyuan Yeh, Jie Jin

**Affiliations:** 1 Department of Urology, Beijing Friendship Hospital, Capital Medical University, 100050 Beijing, China; 2 Department of Gynaecological Oncology, Tianjin Medical University Cancer Institute and Hospital, National Clinical Research Center for Cancer, Key Laboratory of Cancer Prevention and Therapy of Tianjin, Tianjin’s Clinical Research Center for Cancer, Tianjin, China; 3 Department of Urology, Peking University First Hospital, 100034 Beijing, China; 4 Beijing Key Laboratory of Urogenital diseases (male) molecular diagnosis and treatment center, Beijing, China; 5 Department of Urology, Beijing Chaoyang Hospital, Capital Medical University, 100020 Beijing, China; 6 Chawnshang Chang Sex Hormone Research Center, Tianjin Institute of Urology, Tianjin Medical University, 300211 Tianjin, China; 7 George Whipple Lab for Cancer Research, Departments of Pathology, Urology, Radiation Oncology, and The Wilmot Cancer Center, University of Rochester Medical Center, Rochester, NY USA

**Keywords:** Urogenital reproductive disorders, Prostatic diseases, Preclinical research, Endocrine reproductive disorders

## Abstract

Benign prostatic hyperplasia (BPH) is the most common and progressive urological disease in elderly men worldwide. Epidemiological studies have suggested that the speed of disease progression varies among individuals, while the pathophysiological mechanisms of accelerated clinical progression in some BPH patients remain to be elucidated. In this study, we defined patients with BPH as belonging to the accelerated progressive group (transurethral resection of the prostate [TURP] surgery at ≤50 years old), normal-speed progressive group (TURP surgery at ≥70 years old), or non-progressive group (age ≤50 years old without BPH-related surgery). We enrolled prostate specimens from the three groups of patients and compared these tissues to determine the histopathological characteristics and molecular mechanisms underlying BPH patients with accelerated progression. We found that the main histopathological characteristics of accelerated progressive BPH tissues were increased stromal components and prostatic fibrosis, which were accompanied by higher myofibroblast accumulation and collagen deposition. Mechanism dissection demonstrated that these accelerated progressive BPH tissues have higher expression of the CYP19 and G protein-coupled estrogen receptor (GPER) with higher estrogen biosynthesis. Estrogen functions via GPER/Gαi signaling to modulate the EGFR/ERK and HIF-1α/TGF-β1 signaling to increase prostatic stromal cell proliferation and prostatic stromal fibrosis. The increased stromal components and prostatic fibrosis may accelerate the clinical progression of BPH. Targeting this newly identified CYP19/estrogen/GPER/Gαi signaling axis may facilitate the development of novel personalized therapeutics to better suppress the progression of BPH.

## Introduction

Benign prostatic hyperplasia (BPH) is an age-dependent disease, which is present in 20% of men aged 40–50 years, with the prevalence increasing to >80% in men ≥70 years [1]. Histopathologically, BPH is characterized by an increased number of epithelial and stromal cells in the periurethral area of the prostate [2]. BPH is a progressive disease, which is mainly characterized by a deterioration of lower urinary tract symptoms (LUTS) over time, and in some patients with end-stage BPH, such as acute urinary retention and need for BPH-related surgery [3]. The speed of BPH progression varies among individuals [4]. We found that some patients may progress to end-stage BPH at a younger age in their 50s and require surgical treatment, while others may not reach this point until they are in their 70s [5]. Therefore, the goals of therapy for BPH are not only to alleviate LUTS but also to delay the clinical progression of BPH.

The mainstays of current medical therapy to prevent BPH progression include 5α-reductase inhibitors (5ARIs), which reduce dihydrotestosterone (DHT) levels, and α-adrenergic blockers, which lower the adrenergic tone [3]. The results of our previous studies showed that some BPH patients who receive 5ARIs treatment still undergo transurethral resection of the prostate (TURP) due to the clinical progression [6, 7]. Indeed, it has been reported there are still more than 30% of patients suffering from BPH progression after the administration of 5ARIs and α-adrenergic blockers, as many patients either fail to respond or become resistant over time with progression to surgical intervention [8–11]. This suggests that it is particularly important to further reveal the pathophysiological mechanism of BPH progression. Studies have suggested that the imbalance of hormonal homeostasis and inflammation-associated prostatic fibrosis remodels the extracellular matrix to increase prostate tissue stiffness and reduce urethral flexibility [12]. Therefore, in addition to prostatic enlargement and smooth muscle contraction, prostatic fibrosis is considered to be one of the important pathobiological processes associated with accelerated clinical progression of BPH [13].

The imbalance of homeostasis in androgen and estrogen levels with increasing age in prostate tissue plays a pivotal role in the progression of BPH [14–16]. Indeed, estrogen and androgen mediate proliferation, apoptosis, and differentiation in both epithelial cells and stromal cells of the prostate [17]. Although the prostate is commonly considered an androgen-dependent organ, androgen alone may not be sufficient for disease progression. Testosterone can be metabolized via CYP19/aromatase into estrogen [18]. Elevated levels of estrogen in prostate tissue promote hyperplastic growth and abnormal tissue remodeling in BPH progression [19–22]. A recent study revealed there is an androgenic to estrogenic switch in human BPH tissues via increased stromal levels of aromatase [23]. When androgenic pathways are blocked, alternative estrogenic pathways are upregulated to drive the progression of BPH continuously [23]. This suggests that estrogen and its receptor are important factors in the progression of BPH; however, the effect and molecular mechanisms of the estrogen signaling pathway on prostatic fibrosis in patients with accelerated BPH progression remain to be revealed.

In this study, we enrolled prostate specimens from three groups: BPH patients ≤50 years old (defined as early-progressed BPH) or ≥70 years old (defined as elderly BPH) with end-stage clinical progression who require surgery, and bladder cancer patients ≤50 years old who underwent cystoprostatectomy (age-matched control). We intended to identify possible causes of BPH with accelerated progression through histopathological studies. The main histopathological characteristics of BPH tissues with accelerated progression included increased stromal components with a higher ratio of stromal fibrosis, which was accompanied by higher myofibroblast accumulation and collagen deposition. Moreover, mechanism dissection suggested the CYP19/estrogen/G protein-coupled estrogen receptor (GPER)/Gαi signaling pathway may be involved in the regulation of stromal cell proliferation and prostatic fibrosis, which may function to accelerate the clinical progression of patients with BPH.

## Results

### Early-progressed BPH patients are clinically distinct from age-matched controls and elderly BPH patients

To study the characteristics of patients with BPH with different progression speeds, we compared the clinical characteristics among three groups: (1) age ≤50 years old patients who underwent TURP for BPH (early-progressed BPH), vs. (2) age ≤50 years old patients with bladder cancer who underwent cystoprostatectomy (age-matched control), vs. (3) age ≥70 years old patients who underwent TURP for BPH (elderly BPH). The results showed that the elderly BPH group was significantly older (*P* < 0.001), had a larger prostate volume (PV), and had higher prostate-specific antigen (PSA) levels (*P* < 0.001) than the other two groups (Table 1). The early-progressed BPH group had a larger PV than the age-matched control group, but a much smaller PV than the elderly BPH group. There was no significant difference in metabolic parameters among the three groups. However, the BMI in the early-progressed BPH group was higher than in the elderly BPH group (*P* = 0.0253).

**Table 1:** Clinical characteristics of the three groups.

	Age ≤ 50 early-progressed BPH (*n* = 23)	Age-matched control (*n* = 17)	Elderly BPH (*n* = 23)	*P*-value
Age (yr)	47.13 ± 2.69	49.00 ± 4.19	76.57 ± 5.32^b^	<0.001
PV (ml)	41.54 ± 12.33^a^	22.85 ± 8.66	103.19 ± 55.96^b^	<0.001
BMI	25.55 ± 2.33^c^	25.02 ± 2.56	23.79 ± 2.69	0.0705
PSA	1.24 ± 1.08	1.32 ± 1.63	8.04 ± 3.85^b^	<0.001
SBP/DBP, FBG, TG, TCHO, HDL-C, LDL-C				>0.05

^a^There is a statistical difference between the early-progressed BPH group and the age-matched control group.

^b^There is a statistical difference between the three groups.

^c^There is a statistical difference between the early-onset BPH group and the elderly BPH group.

### Increased stromal components and prostatic fibrosis in the early-progressed BPH group

**Fig. 1:**
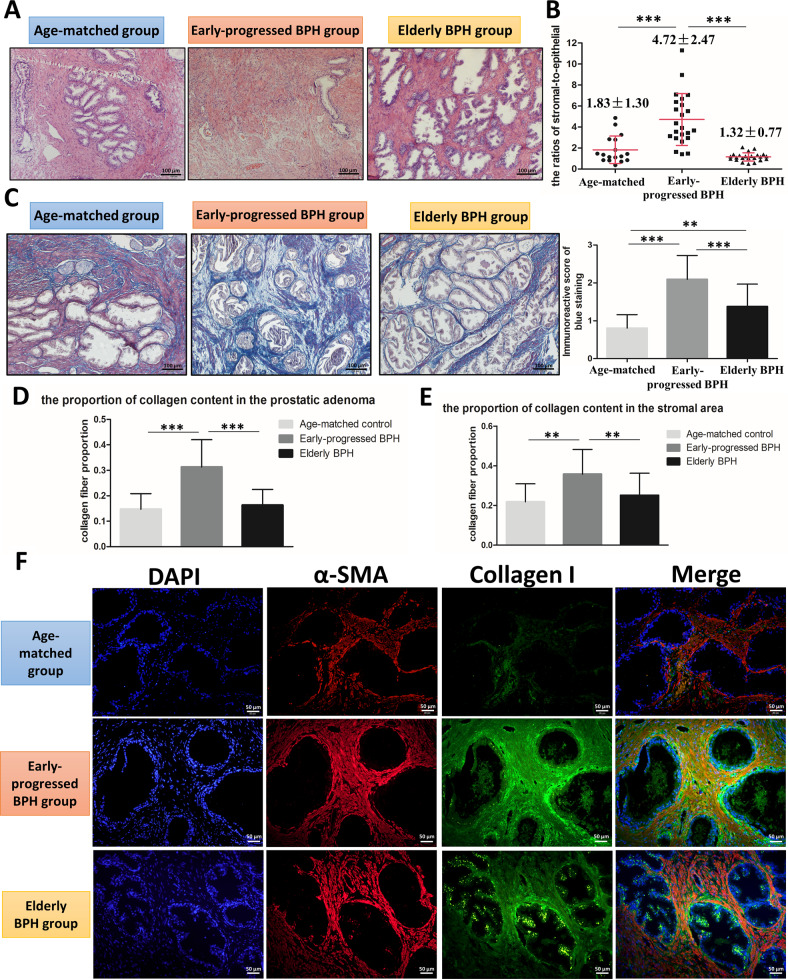
Histological analysis of prostatic specimens from the three groups. **A** H&E staining of paraffin-embedded prostatic tissue specimens from the three groups of patients. Scale bar: 100 μm. **B** Computer image analysis for the ratio of stromal-to-epithelial hyperplasia in the three groups. The variance was similar between the groups. ***P < 0.001. **C** Masson’s trichrome staining for prostatic tissue specimens from the patients of three groups. Scale bar: 100 μm. Right bar graph is the IOD of collagen fiber with blue staining. The variance was similar between the groups. **P < 0.01, ***P < 0.001. **D, E** Computer image analysis of Masson’s trichrome staining and the ratio of the stromal-to-epithelial area. The proportion of collagen fiber in the total prostatic adenoma (**D**) and in the stromal area (**E**) of the three groups. The variance was similar between the groups. **P < 0.01, ***P < 0.001. **F** Prostatic tissues from the three groups of patients were stained for α-SMA (red) and collagen I (green), and the nuclei were counterstained with DAPI (blue) by immunofluorescence. Merged images indicate a myofibroblastic phenotype. Scale bar: 50 μm.

To investigate the difference in histopathology among the three groups, we first performed hematoxylin and eosin (H&E) staining. The results revealed increased stromal components in the early-progressed BPH group compared to the other two groups (Fig. 1A). Importantly, the whole slide image analysis for all available prostate specimens revealed the ratio of stromal-to-epithelial hyperplasia in the early-progressed BPH group was significantly higher than in the other two groups (age-matched control group: 1.83 ± 1.30, early-progressed BPH group: 4.72 ± 2.47, elderly BPH group: 1.32 ± 0.77, *P* < 0.001; Fig. 1B).

In addition, we investigated prostatic fibrosis, which is considered an important characteristic of LUTS related to BPH [13]. We assayed the collagen content in prostate specimens by Masson’s trichrome staining [24]. Quantification of the intensity of collagen content within prostatic tissue areas showed that the immunoreactive score (IOD) of total blue intensity was significantly different among the age-matched control group (0.81 ± 0.36), early-progressed BPH group (2.09 ± 0.63), and elderly BPH group (1.38 ± 0.59) (Fig. 1C). Based on the Masson staining results, Fig. 1D, E showed the proportion of collagen fibers in the early-progressed BPH group was significantly higher than the other two groups in both total prostatic area and prostatic stromal area (total area at 14.72 ± 6.13% vs. 31.30 ± 10.74% vs. 16.34 ± 6.12%, *P* < 0.001, and stromal area at 21.85 ± 9.10% vs. 35.76 ± 10.25% vs. 25.12 ± 11.10%, *P* < 0.01).

To further demonstrate increased prostatic fibrosis in early-progressed BPH patients, immunofluorescence studies were conducted to examine the key markers of myofibroblasts, collagen I, and α-SMA. The results revealed the expression of α-SMA and collagen I was higher in the early-progressed BPH group than in the other two groups (Fig. 1F). Moreover, it also showed that the early-progressed BPH group exhibited higher expression of colocalized collagen I and α-SMA protein, suggesting increased myofibroblast phenoconversion in these patients (Fig. 1F). We also examined the expression of TGF-β1, which can promote fibrosis through the transcription regulation of α-SMA and collagen I [25]. The results revealed the control group tissues displayed mild expression, while the elderly BPH group showed strong expression of TGF-β1, which was predominantly restricted to the epithelium. However, TGF-β1 expression was significantly increased in both the epithelium and the stromal area in the early-progressed BPH samples (Supplementary Fig.1A).

Collectively, histopathological studies suggest that the prostatic tissues of BPH patients with accelerated clinical progression have higher stromal components and increased prostatic fibrosis with higher collagen deposition and myofibroblast accumulation.

### Increased estrogen promotes prostatic fibrosis in early-progressed BPH patients

**Fig. 2:**
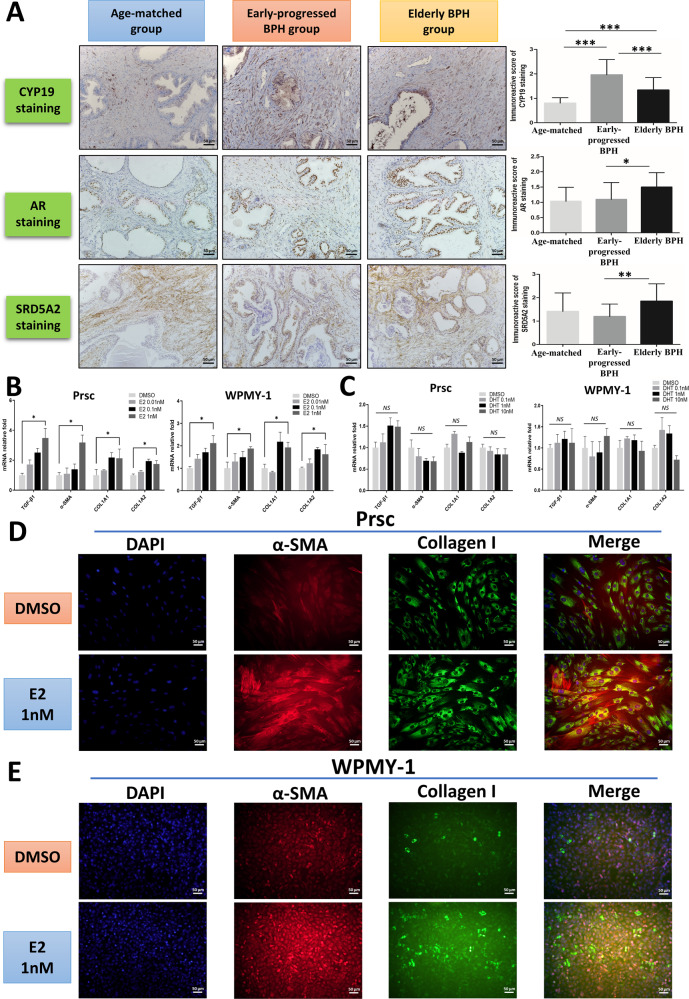
Increased estrogen promotes prostatic fibrosis in early-progressed BPH patients. **A** IHC staining for SRD5A2, AR, and CYP19 in the prostatic tissue specimens from the three groups. Scale bar: 50 μm. Right bar graph is the IOD of positive SRD5A2, AR, and CYP19 staining. The variance was similar between the groups. *P < 0.05, **P < 0.01, ***P < 0.001. **B, C** Prsc and WPMY-1 were treated with E2 (**B**) at 0 nM (DMSO), 0.01 nM, 0.1 nM, and 1 nM, or with DHT (**C**) at 0 nM, 0.1 nM, 1 nM, and 10 nM for 48 h. Q-PCR analysis was used to detect the expression of TGF-β1, α-SMA, COL1A1, and COL1A2. The variance was similar between the groups. *P < 0.05; NS non-significant. **D, E** Immunofluorescence staining for α-SMA, collagen I, and DAPI in Prsc (**D**) and WPMY-1 (**E**) with or without 1 nM E2 treatment for 72 h. Merged images indicate a myofibroblastic phenotype. Scale bar: 50 μm.

Early studies have documented that the imbalance of homeostasis in androgen and estrogen levels could promote prostatic stromal cell proliferation and may change the morphological characteristics of the prostatic tissues [26, 27]. In the above histopathological studies, we hypothesized that androgen or estrogen signaling may involve in the regulation of histopathological changes in the prostate of BPH patients with accelerated clinical progression. Therefore, we examined the expression of steroid-5α reductase type 2 (SRD5A2), androgen receptor (AR), and CYP19 in prostatic tissues by immunohistochemistry (IHC) staining. The results revealed the expression of SRD5A2 and AR in the early-progressed BPH group was slightly lower than in the other two groups (Fig. 2A). In contrast, higher CYP19 expression was found in the early-progressed BPH group in both the epithelium and stroma, which was confirmed by the IOD analysis of CYP19 intensity (*P* < 0.001, Fig. 2A). This result suggests that more androgen can be converted to estrogen, and the elevated estrogen levels may be involved in the pathologic modification of prostatic stroma in patients with BPH with accelerated clinical progression.

To further study the impact of androgen and estrogen on the prostatic stromal cells, we applied early-progressed BPH primary stromal cells (Prsc) and the prostatic stromal cell line, WPMY-1, to assay the effects of DHT or estradiol (E2) on fibrosis gene expression. The results of Q-PCR (Fig. 2C) and western blot (Supplementary Fig. 1B) revealed DHT could not regulate the expression of fibrosis genes; however, 1 nM E2 significantly increased the expression of fibrosis genes (TGF-β1, α-SMA, COL1A1, and COL1A2) in both Prsc and WPMY-1 (Fig. 2B). Importantly, immunofluorescence studies in both Prsc (Fig. 2D) and WPMY-1 (Fig. 2E) revealed 1 nM E2 increased myofibroblast accumulation, with higher expression of colocalized collagen I and α-SMA protein.

### Estrogen promotes prostatic fibrosis in early-progressed BPH patients by activating GPER/Gαi signaling

**Fig. 3:**
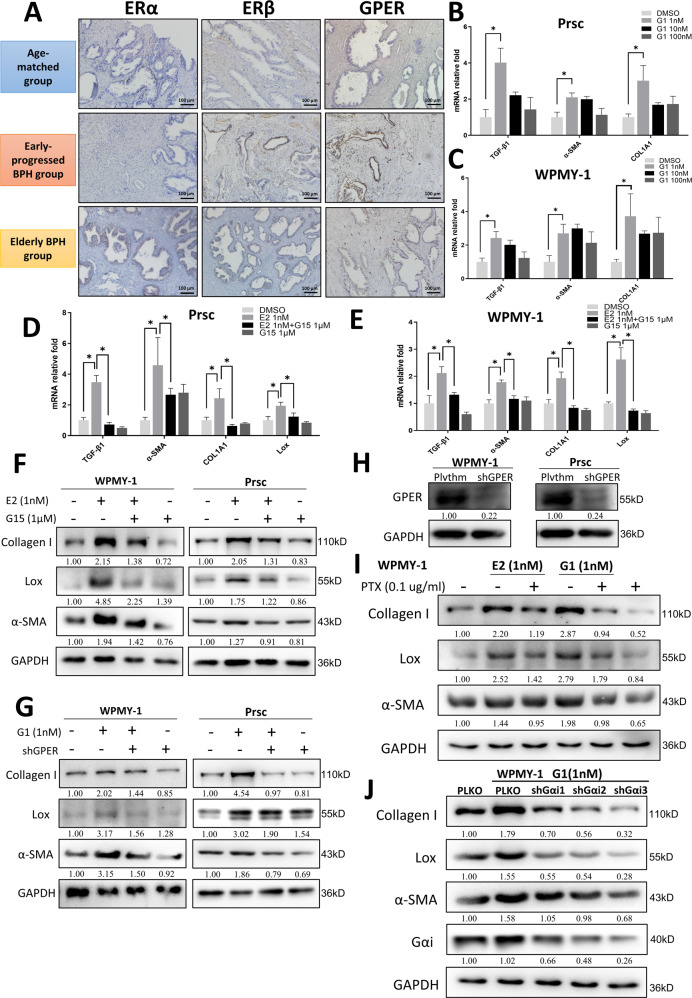
Estrogen promotes prostatic fibrosis in early-progressed BPH patients by activating GPER/Gαi signaling. **A** IHC staining for ERα, ERβ, and GPER in prostatic tissue specimens from the three groups. Scale bar: 100 μm. **B, C** Prsc (**B**) and WPMY-1 (**C)** were treated with G1 at 0 nM (DMSO), 1 nM, 10 nM, and 100 nM for 48 h. Q-PCR analysis was used to detect the expression of TGF-β1, α-SMA, and COL1A1. The variance was similar between the groups. *P < 0.05. **D, E** Prsc (**D**) and WPMY-1 (**E**) were treated with or without 1 nM E2 in the presence or absence of 1 µM G15 for 48 h, and TGF-β1, α-SMA, COL1A1, and Lox were analyzed by Q-PCR. The variance was similar between the groups. *P < 0.05. **F, G** WPMY-1 and Prsc were treated with 1 nM E2 plus 1 µM G15 (**F**) or 1 nM G1 plus shGPER (**G**) for 72 h, and collagen I, α-SMA, and Lox were detected by western blot. **H** The knockdown efficiency of shGPER in WPMY-1 and Prsc. **I** WPMY-1 were treated with 1 nM E2 or 1 nM G1 plus PTX at 0.1 µg/ml for 72 h, and collagen I, α-SMA, and Lox were detected by western blot. **J** WPMY-1 were treated with 1 nM G1 and were transduced with or without the three subunits of Gαi-shRNA for 72 h, and collagen I, α-SMA, Lox, and Gαi were detected by western blot. GAPDH was used as a loading control.

Biological responses to estrogens are mainly mediated by the classical ERα and/or ERβ [28]. Recent studies have indicated that estrogens may function *via* the membrane estrogen receptor, GPER, to mediate their estrogenic effects in normal and malignant cell contexts, including in prostatic stromal cells [29, 30]. To further dissect which type of estrogen receptor (ER) is involved in the estrogen-promoted prostatic fibrosis of BPH patients with accelerated progression, we examined the expression of ERα, ERβ, and GPER in the three groups. The results showed the expression of GPER was higher in the early-progressed BPH group in both the epithelium and stroma (Fig. 3A). In contrast, there were no significant changes in the expression of ERα and ERβ among the three groups. Results from either knocking down or overexpressing ERα or ERβ revealed few changes in the expression of fibrosis genes in Prsc and WPMY-1 (data not shown).

We then examined the role of GPER in prostatic fibrosis. The cells were treated with G1 (an agonist of GPER) [31] at concentrations of 0 (DMSO), 1 nM, 10 nM, and 100 nM. Q-PCR analysis revealed 1 nM G1 significantly increased the expression of some fibrosis genes (TGF-β1, α-SMA, and COL1A1) in both Prsc (Fig. 3B) and WPMY-1 (Fig. 3C). In contrast, adding 1 µM G15 (an antagonist of GPER) [31] reversed the E2-induced fibrosis genes in both Prsc (Fig. 3D) and WPMY-1 (Fig. 3E). The western blot also showed that G15 (Fig. 3F) or knockdown (shGPER) (Fig. 3G) reversed the E2- or G1-induced collagen I, Lox, and α-SMA expression in both Prsc and WPMY-1. Figure 3H showed the knockdown efficiency of shGPER.

Previous studies have reported that the estrogen-activated GPER regulates the conduction of downstream signaling pathways through the G protein, Gαi [32, 33]. To further dissect the mechanism of estrogen/GPER function through which G proteins impact prostatic fibrosis, we treated cells with the Gαi inhibitor, pertussis toxin (PTX), at 0.1 µg/ml [34]. Western blot showed that PTX significantly inhibited the E2- or G1-induced fibrosis genes in WPMY-1 (Fig. 3I), indicating Gαi mediates the estrogen/GPER signals to increase the fibrosis genes. Importantly, we also constructed three shRNAs to knock down three subunits of Gαi (shGαi1, shGαi2, shGαi3) [35], and the results revealed all three shRNAs inhibited G1-induced collagen I, Lox, and α-SMA expression, especially knockdown of shGαi3 (Fig. 3J).

### Estrogen/GPER/Gαi increases prostatic fibrosis in early-progressed BPH patients by altering HIF-1α expression

To further investigate the intracellular signals that mediate estrogen/GPER/Gαi signaling, we focused on HIF-1α, as recent reports have indicated that HIF-1α is downstream of GPER in several diseases [36–38]. Moreover, a previous study found that HIF-1α promoted fibrogenesis by facilitating epithelial-mesenchymal transition (EMT) or increasing the expression of extracellular matrix (ECM) remodeling factors and lysyl oxidase genes [39].

**Fig. 4:**
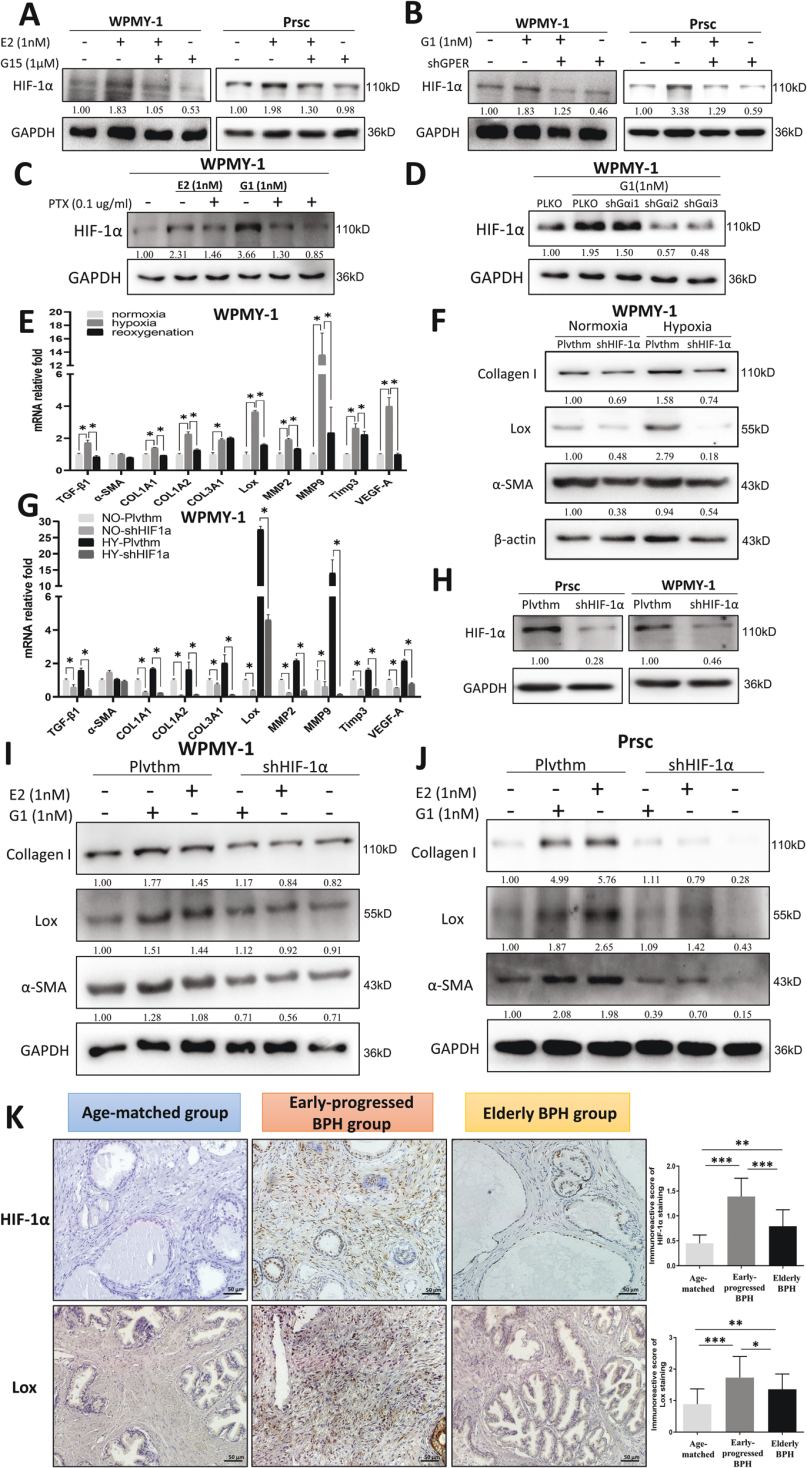
Estrogen/GPER/Gαi increases prostatic fibrosis in early-progressed BPH patients by altering HIF-1α expression. **A, B** WPMY-1 and Prsc were treated with 1 nM E2 plus 1 µM G15 (**A**) or 1 nM G1 and transduced with or without shGPER (**B**) for 72 h, and the expression of HIF-1α was detected by western blot. **C** WPMY-1 were treated with 1 nM E2 or 1 nM G1 plus PTX at 0.1 µg/ml for 72 h, and HIF-1α expression was detected by western blot. **D** WPMY-1 were treated with 1 nM G1 and were transduced with or without the three subunits of Gαi-shRNA for 72 h, and HIF-1α expression was detected by western blot. **E** WPMY-1 were subjected to normoxia, hypoxia, and reoxygenation conditions, and the indicated genes were analyzed by Q-PCR, *P < 0.05. **F, G** Plvthm-WPMY-1 or shHIF-1α-WPMY-1 were subjected to hypoxia (HY) or normoxia (NO) for 48 h, and the indicated genes were analyzed by western blot (**F**) and Q-PCR (**G**), *P < 0.05. **H** The knockdown efficiency of shHIF-1α in Prsc and WPMY-1. **I, J** Plvthm/shHIF-1α-WPMY-1 (**I**) or Plvthm/shHIF-1α-Prsc (**J**) were treated with vehicle control, 1 nM E2, or 1 nM G1 for 72 h, and the indicated genes were analyzed by western blot. **K** IHC staining for HIF-1α and Lox in prostatic tissue specimens from the three groups. Scale bar: 50 μm. Right bar graph is the IOD of positive HIF-1α and Lox staining. The variance was similar between the groups. *P < 0.05, **P < 0.01, ***P < 0.001. GAPDH or β-actin was used as the loading control in all western blots.

We performed a western blot to detect the protein level of HIF-1α after treatment with E2 or G1. The results showed that E2 or G1 increased the expression of HIF-1α, whereas treatment with G15 (Fig. 4A) or transduction with shGPER (Fig. 4B) reversed this regulation in both WPMY-1 and Prsc. Consistent with this, treating with PTX reversed the E2- or G1-induced HIF-1α expression (Fig. 4C), and the three Gαi shRNAs reversed the G1-induced HIF-1α expression in WPMY-1 (Fig. 4D). Importantly, we found that hypoxia (HY) increased the expression of fibrosis genes (TGF-β1, α-SMA, COL1A1, COL1A2, COL3A1, and Lox), ECM remodeling genes (MMP2, MMP9, and Timp3), and VEGF-A in WPMY-1 compared to normoxic (NO) conditions (Fig. 4E–G). The reoxygenation condition (reintroducing cells to normoxic conditions following hypoxia) (Fig. 4E) or lentiviral transduction with HIF-1α-shRNA (Fig. 4F, G) reversed the upregulation of hypoxia-induced genes. We also noticed that transducing shHIF-1α suppressed these genes not only under hypoxia but also under normoxic conditions (Fig. 4F, G). Figure 4H showed the knockdown efficiency of shHIF-1α. In addition, western blot results from an interruption approach by knocking down HIF-1α also revealed shHIF-1α may partially reverse E2- or G1-induced expressions of collagen I, Lox, and α-SMA in both WPMY-1 (Fig. 4I) and Prsc (Fig. 4J). Next, we verified the expression of HIF-1α and Lox in tissue samples by IHC staining. The results revealed the expression of HIF-1α and Lox in the prostatic stromal cells of early-progressed BPH patients was significantly higher than in the other two groups (Fig. 4K).

### Estrogen/GPER/Gαi signaling increases HIF-1α expression by altering the protein stability of HIF-1α

To further dissect the molecular mechanism underlying how estrogen/GPER/Gαi signaling increases HIF-1α expression, we found that adding E2 or G1 led to few changes in HIF-1α expression at the mRNA level (data not shown). Therefore, we hypothesized that post-transcriptional regulation may affect the change in HIF-1α protein expression.

**Fig. 5:**
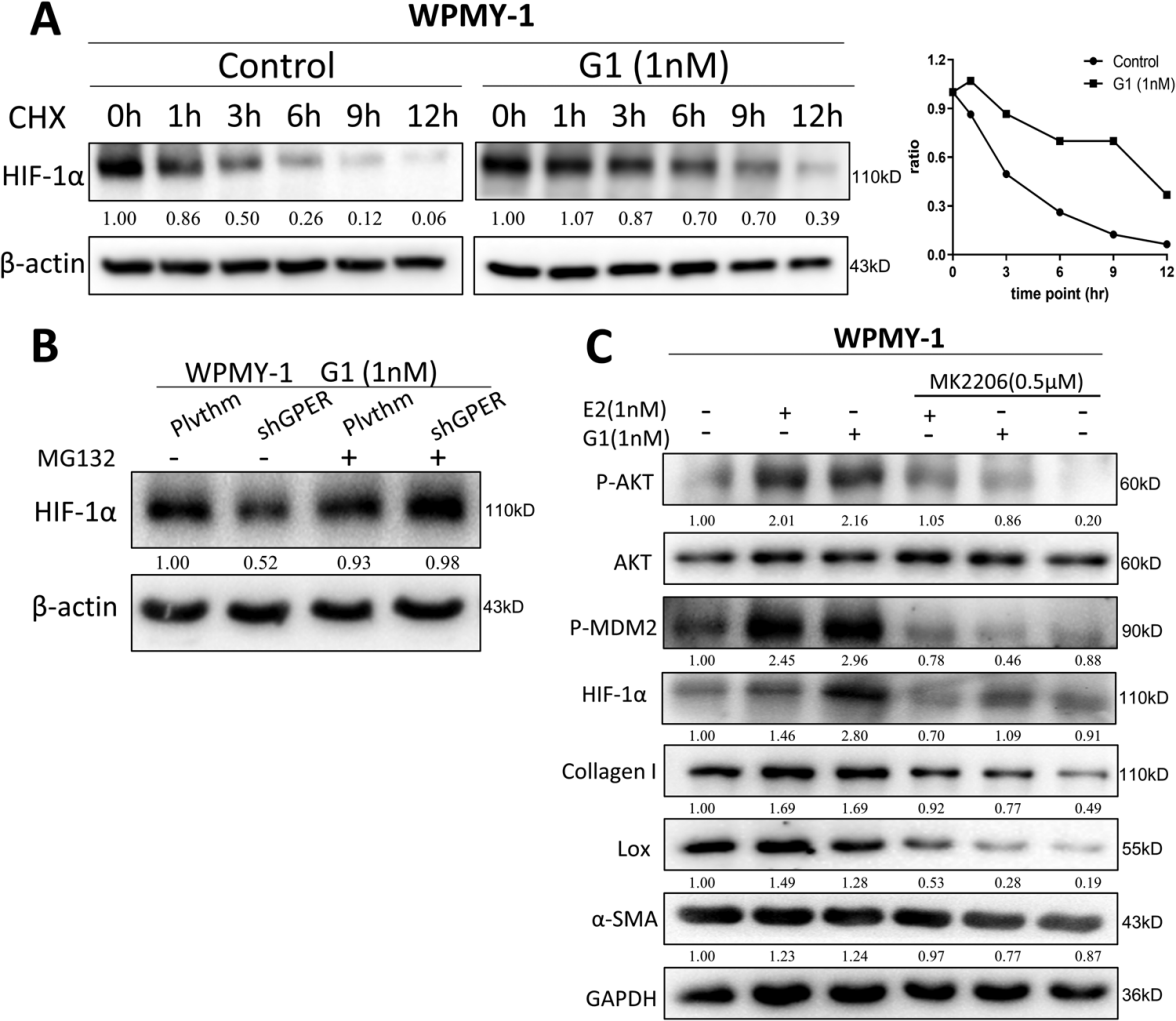
Estrogen/GPER/Gαi signaling increases HIF-1α expression by altering protein stability of HIF-1α. **A** WPMY-1 were pretreated with 10 mg/ml cycloheximide (CHX) and then treated with vehicle control or 1 nM G1 for 0–12 h. HIF-1α protein levels were analyzed by western blot. The right graph is the quantified HIF-1α degradation curve. **B** Plvthm/shGPER-WPMY-1 were treated with or without MG132 (5 mM) for 3 h in the presence of 1 nM G1, and then HIF-1α protein levels were analyzed by western blot. **C** WPMY-1 were treated with 1 nM E2 or 1 nM G1 with or without 0.5 µM MK2206 for 72 h, and the expression of the indicated genes was detected by western blot. GAPDH or β-actin was used as the loading control.

As HIF-1α protein synthesis is mainly regulated by protein stability [40], we examined HIF-1α protein stability in the presence of 10 ug/ml cycloheximide (CHX) with or without G1 treatment. The results revealed the degradation rate of HIF-1α protein was lower under G1 treatment than the control group (Fig. 5A), suggesting that G1 promotes HIF-1α protein stability. Importantly, the shGPER-reduced HIF-1α expression in response to G1 treatment was partially reversed by treatment with the proteasome inhibitor MG132. Additionally, MG132 may not further rescue/reverse HIF-1α expression under G1 treatment (Fig. 5B), indicating G1 suppressed HIF-1α degradation in a proteasome-dependent manner.

Previous reports have shown that activation of the PI3K/AKT signaling pathway can increase the phosphorylation of MDM2, thereby regulating the stabilization of HIF-1α [41, 42]. Other studies have also revealed estrogen/GPER signaling can activate the PI3K/AKT signaling pathway [43–46]. We found that treatment with E2 or G1 phosphorylated AKT and MDM2 (Fig. 5C), while the AKT inhibitor, MK2206, could reverse the E2- or G1-induced expression of P-AKT, P-MDM2, HIF-1α, collagen I, Lox, and α-SMA, suggesting that the profibrotic effects of estrogen/GPER/Gαi signaling functions via altering intracellular PI3K/AKT/P-MDM2 signaling to impact the protein stability of HIF-1α.

### Estrogen/GPER/Gαi signaling increases prostatic stromal cell proliferation by altering EGFR/ERK signaling

**Fig. 6:**
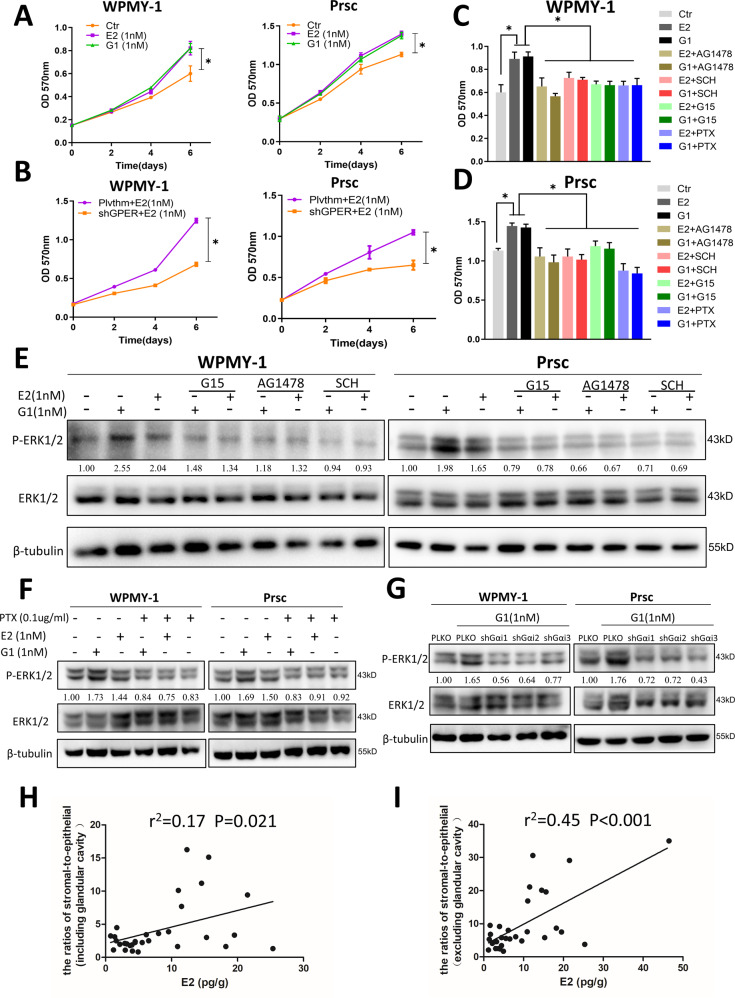
Estrogen/GPER/Gαi signaling increases prostatic stromal cell proliferation by altering EGFR/ERK signaling. **A** MTT assays were performed using WPMY-1 (left) cells and Prsc (right) treated with DMSO (Ctr), 1 nM E2, or 1 nM G1 on days 2, 4, and 6. The variance was similar between the groups. *P < 0.05. **B** MTT assays using Plvthm/shGPER-WPMY-1 (left) and Plvthm/shGPER-Prsc (right) in the presence of 1 nM E2 on days 2, 4, and 6. The variance was similar between the groups. *P < 0.05. **C, D** WPMY-1 (**C**) and Prsc (**D**) were treated with DMSO (Ctr), 1 nM E2, 1 nM G1, or 1 nM E2/1 nM G1 with 0.5 μM AG1478, 10 nM SCH772984, 1 μM G15, or 0.1 µg/ml PTX. MTT assays were performed after 6 days culture. The variance was similar between the groups. *P < 0.05. **E** WPMY-1 (left) and Prsc (right) were treated with DMSO (Ctr), 1 nM E2, 1 nM G1, or 1 nM E2/1 nM G1 with 0.5 μM AG1478, 10 nM SCH772984, or 1 μM G15 for 12 h. The expression of ERK1/2 and P-ERK1/2 was detected by western blot. **F** WPMY-1 (left) and Prsc (right) were treated with DMSO (Ctr), 1 nM E2, 1 nM G1, or 1 nM E2/1 nM G1 with 0.1 µg/ml PTX for 12 h. The expression of ERK1/2 and P-ERK1/2 was detected by western blot. **G** PLKO**-**WPMY-1 (left) and PLKO-Prsc (right) were treated with DMSO (Ctr), 1 nM G1, or 1 nM G1 with transducing cells with the three subunits of Gαi shRNAs. The expression of ERK1/2 and P-ERK1/2 was detected by western blot. β-tubulin was used as a loading control in all western blots. **H, I** Correlation analysis between prostatic estrogen concentrations and the ratio of the stromal-to-epithelial area (**H:** epithelial area including or **I:** excluding the glandular cavity area) to the prostate was determined by Pearson correlation analysis. LC-MS/MS was used to examine the E2 concentrations in the prostatic tissues of 31 patients with BPH.

Previous studies have indicated estrogen could go through GPER to activate EGFR/ERK in prostatic stromal cells [26, 47]. We found that treatment with E2 or G1 promoted the growth of WPMY-1 and Prsc on days 4 and 6 using MTT assay (Fig. 6A), and that transducing shGPER suppressed stromal cell proliferation in the presence of E2 (Fig. 6B). This finding confirmed that estrogen could function through GPER to promote the proliferation of prostatic stromal cells.

Next, we used signaling pathway inhibitors to clarify the potential downstream pathways of estrogen/GPER. The cells treated with the EGFR inhibitor AG1478, the ERK inhibitor SCH772984, the GPER antagonist G15, and the Gαi inhibitor PTX all revealed these inhibitors could partially reverse E2- or G1-induced stromal cell proliferation on day 6 in both WPMY-1 (Fig. 6C) and Prsc (Fig. 6D). Moreover, western blot showed that the phosphorylation of ERK1/2 induced by E2 or G1 could also be suppressed by AG1478, SCH772984, G15 (Fig. 6E), and PTX (Fig. 6F). Consistent with this, the three Gαi shRNAs could also reverse the G1-induced P-ERK1/2 expression in prostatic stromal cells (Fig. 6G). We also conducted liquid chromatography-tandem mass spectrometry (LC-MS/MS) to detect the estrogen levels in prostatic tissues of 31 patients with BPH. The results revealed a positive correlation between estrogen level and the ratio of stromal-to-epithelial hyperplasia (epithelial area including or excluding the glandular cavity area) in the prostate (Fig. 6H, I), suggesting that estrogen plays a critical role in the increased stromal components in the prostate.

## Discussion

Although prostatic enlargement correlates with increased urethral resistance and LUTS, many men experience bladder outlet obstruction and LUTS in the absence of significant prostatic enlargement [48]. Among the three groups of patients enrolled in this study, some prostatic tissues of patients from the control group had asymptomatic pathological BPH. However, the prostate resections for this group were due to bladder cancer, and not the clinical progression of BPH. Both the early-progressed BPH group and the elderly BPH group had end-stage clinical BPH requiring surgical treatment; however, the two groups differed in that they had different rates of progression to the end-stage BPH. The early-progressed BPH patients are extreme cases of BPH progression, so we chose to compare this group of patients to the normal-speed progressive patients to highlight the factors that may contribute to rapid BPH progression. Although the prostate volume in the early-progressed BPH group was larger than in the control group, it was still smaller than in the elderly BPH group. Therefore, this finding suggests that the acceleration of BPH progression could be due to the histopathological changes in the prostate. Three major pathobiological processes, including prostatic enlargement, smooth muscle contraction, and prostatic fibrosis, can act either independently or in combination to promote the clinical progression of BPH. Recently, prostatic fibrosis was deemed to be an important pathophysiological process associated with BPH/LUTS [13]. It has been reported that highly collagenous tissue of the prostate may decrease urethral compliance and elastin expression, which can increase urethral pressure [13, 24, 49–52]. In BPH patients with accelerated clinical progression, prostatic fibrosis causes urethral resistance and dysfunction of prostate tissue that cannot be “relaxed” with α-adrenergic blockers. Furthermore, the proliferation and fibrogenesis of the prostatic stroma may result in an increase in prostate volume rather than glandular hyperplasia. Thus, 5ARIs may be ineffective for these patients. Accordingly, our results suggest BPH secondary to stromal hyperplasia with prostatic fibrosis is considered a primary cause of accelerated clinical progression of BPH.

As serum androgens decline with advancing age, estrogens remain relatively constant, with an increased serum E2 to T ratio [15]. The imbalance of androgen and estrogen homeostasis is associated with the development of BPH/LUTS [14]. A recent study revealed the existence of an androgenic to estrogenic switch in the progression of BPH, which is facilitated via increased stromal levels of aromatase [23]. In men, the majority of circulating estrogen is formed from the aromatization of testosterone, mainly in fat and muscle [53]. Serum estrogens are associated with prostate volume and other types of prostatic histological remodeling in BPH patients [54, 55]. However, the effects of androgen and estrogen homeostasis on the speed of BPH progression remain largely unknown. Here, we found that aromatase was highly expressed in the accelerated progressive BPH tissues, whereas SRD5A2 and AR were weakly expressed. These results suggest that the androgenic to estrogenic switch also exists in BPH tissues with accelerated progression. We also noticed that the early-progressed BPH group had a higher BMI, which is associated with high expression and activity of aromatase and increased circulating estrogen levels [18, 56, 57]. Coincidentally, many studies have reported that a higher BMI with metabolic syndrome is associated with BPH/LUTS development and progression [58–60]. Therefore, we hypothesized that high expression of aromatase may increase estrogen/ER signaling, which may be involved in the regulation of histopathological changes in the prostate of BPH patients with accelerated clinical progression.

Park et al. found that stromal cells isolated from young organ donors predominantly used GPER to regulate cell growth, but cells isolated from elderly BPH patients used classical ER signaling (ERα, ERβ) to regulate cell growth [26]. In this study, we found only the expression of GPER was higher in the early-progressed BPH group. Mechanism dissection revealed estrogen may not function through ERα or ERβ; however, the estrogen/GPER/Gαi signaling could promote prostatic stromal cell proliferation and fibrosis. This indicates estrogen/GPER is the key factor for the increase in stromal components and fibrosis process of the prostate in accelerated clinical progressive BPH patients.

Although previous studies suggest HIF-1α and EGFR/ERK signaling may be the targets of GPER [26, 36–38, 47], we have demonstrated for the first time in prostate stromal cells that estrogen/GPER/Gαi functions through PI3K/AKT/P-MDM2 to promote HIF-1α stability, which in turn promotes prostate fibrosis with TGF-β1. The increased expression of HIF-1α was confirmed in prostatic stromal tissues of early-progressed BPH patients, as well as elevated expressions of fibrosis genes, such as α-SMA, collagen I, TGF-β1, and Lox. Moreover, we used multiple inhibitors to verify that estrogen promoted the proliferation of prostatic stromal cells by activating the GPER/Gαi/EGFR/ERK signaling pathway. A positive correlation between prostatic estrogen level and the ratio of the stromal-to-epithelial area was also confirmed in 31 ungrouped patients with BPH.

**Fig. 7:**
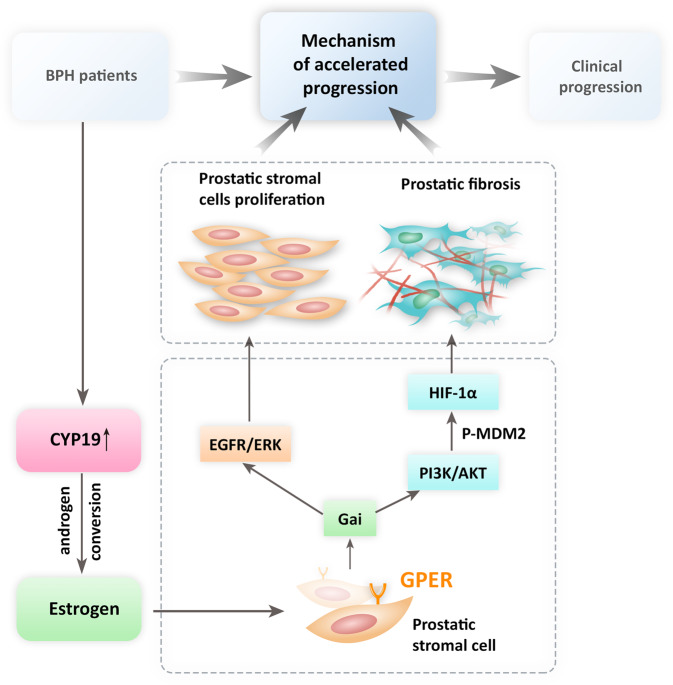
Schematic depiction and molecular mechanisms of early rapid clinical progression of patients with BPH. The CYP19/estrogen/GPER/Gαi pathway triggers the early rapid clinical progression of patients with BPH by promoting prostatic fibrosis and prostatic stromal cell proliferation.

Taken together, our results describe the prostatic histopathologic characteristics of patients with BPH with accelerated progression. We found the increased stromal components, and prostatic fibrosis may be the key factor for the rapid clinical progression of BPH/LUTS. The CYP19/estrogen/GPER pathway modulates EGFR/ERK and HIF-1α/TGF-β1 signaling, all of which have key roles in the proliferation and fibrogenesis of prostatic stromal cells in patients with accelerated clinical progression of BPH (Fig. 7). However, not restricted to early-progressed BPH patients, prostatic fibrosis also can act independently or in combination with prostatic enlargement and smooth muscle contraction to promote clinical progression of BPH/LUTS in men at any age. These findings may provide personalized medicine therapeutic targets for stromal hyperplasia with prostatic fibrosis. In the future, antifibrotic treatment by targeting the CYP19/estrogen/GPER signaling pathway combined with current drugs may be effective in delaying the progression of BPH patients with prostatic fibrosis [6, 8, 61].

## Materials and methods

### Patients and prostatic tissues

The patients were selected from the electronic medical record system of BPH patients who underwent TURP, or bladder cancer patients who underwent cystoprostatectomy in the Department of Urology, Beijing Friendship Hospital, and Peking University First Hospital, Beijing, China.

Prostatic tissues were collected from 63 patients grouped according to their age, diagnoses, and medical history: (1) the early-progressed BPH group, which consisted of 23 patients (age ≤50 years old) with end-stage clinical BPH who underwent TURP; (2) age-matched control group, which consisted of 17 age ≤50 years old patients with bladder cancer who underwent cystoprostatectomy; and (3) elderly BPH group, which consisted of 23 patients (age ≥70 years old) with end-stage clinical BPH who underwent TURP. All of the patients enrolled in this study had been medicated with either 5ARIs or α-adrenergic blockers for no longer than 6 months before surgery. Before surgery, the prostate volume of the patients was investigated by transrectal ultrasonography of the prostate. Urine routine, uroflowmetry, urodynamic examination, cystoscopy, and residual urine were also examined before surgery. Patients who had urinary tract infection, prostatitis, dysfunctional voiding, bladder dysfunction, bladder neck obstruction, urethral stricture, cystolith, previous prostate-related surgery, or a history of urethral catheterization were excluded from this study. The prostate specimens were examined microscopically by two pathologists to determine a diagnosis of BPH without prostate cancer, prostatic intraepithelial neoplasia, and prostatic stromal sarcoma.

### BPH primary stromal cell and stromal cell line culture

BPH tissues were obtained from patients who underwent TURP and were aged ≤50 years old. Prostatic stromal cells were generated from each specimen. During the surgery, the prostates were minced with a sterile scalpel into 1 mm^3^ fragment and then subjected to enzymatic dissociation in RPMI-1640 media (Invitrogen, Grand Island, NY, USA) supplemented with 10% fetal bovine serum (FBS), 200 U/ml collagenase type I, and 100 U/ml DNase type I. Tissue dissociation was accomplished using a magnetic stir bar to provide gentle agitation for 2–4 h at 37 °C. The resulting cell suspension was then washed twice with phosphate-buffered saline (PBS), resuspended in PBS, and layered over a 5–10% discontinuous Percoll gradient to isolate stromal cells, which were used to establish primary cell cultures. The cells were maintained in DMEM-F12 media supplemented with 15% FBS, 5 ng/ml EGF, and 1% penicillin/streptomycin in a standard 37 °C humidified incubator with 5% CO_2_. The primary stromal cells used in all experiments were cultured for less than six passages and were confirmed to have a fibroblast phenotype by immunofluorescence (positive vimentin expression and negative Epcam [epithelial cell marker] expression) (Supplementary Fig. 2).

The human prostatic stromal cell line WPMY-1 was purchased from American Type Culture Collection (ATCC, Manassas, VA, USA) and cultured in DMEM (Invitrogen) supplemented with 10% FBS, penicillin (25 units/ml), streptomycin (25 g/ml), and 1% L-glutamine. All cell lines were cultured in a standard 37 °C humidified incubator with 5% CO_2_. Hypoxia was achieved by maintaining the cells at 1% O_2_, 5% CO_2_, and 94% N_2_ in a hypoxic chamber (Coy Laboratory Products) with oxygen sensor controls, and with temperature (37 °C), humidity, CO2, and N2 gas regulators.

To eliminate the interference of other steroid hormones, all of the reagent treatments for cell cultures were performed in charcoal-dextran stripped FBS media.

### Histopathological analysis

The whole slide image analysis was performed by the APERIO AT2 system (Leica Biosystems, Wetzlar, Germany). The detailed procedure for determining the ratio of stromal-to-epithelial area and collagen content in the prostate is presented in Supplementary Fig.3. Positive staining for cells and tissues was analyzed using the Image-Pro Plus 6.0 software (Media Cybernetics, Rockville, MD, USA) as the average immunoreactive score (IOD) in 10 random fields at ×200 magnification. We use double-blind strategy in the histopathological analysis experiments.

### Reagents and materials

α-SMA (7817), collagen I (34710), TGF-β1 (92486), AR (74272), CYP19 (18995), ERα (32063), ERβ (288), GPER (39742), and Lox (174316) antibodies were purchased from Abcam (Cambridge, UK). Gαi (5290), P-AKT (4060), AKT (4685), P-MDM2 (3521), ERK1/2 (4695), P-ERK1/2 (4370), GAPDH (2118), β-actin (3700), and β-tubulin (2128) antibodies were purchased from Cell Signaling Technology (Boston, MA, USA). SRD5A2 (SAB2105567) and HIF-1α (SAB5200017) antibodies, estradiol, dihydrotestosterone, cycloheximide, and MG132 were purchased from Sigma–Aldrich (Darmstadt, Germany). Anti-mouse/rabbit second antibody for western blot was from Invitrogen. G1, G15, AG1478, SCH772984, and PTX were purchased from Cayman (Ann Arbor, MI, USA).

### Estrogen level quantification

Thirty-one ungrouped fresh prostatic tissues of patients with BPH were harvested during the TURP. The E2 prostatic tissue concentrations were quantified using the LC-MS/MS method. Details of the LC-MS/MS procedure for tissue hormone quantification have been reported previously [62]. The estrogen content of each sample was measured by the concentrations of standard E2.

### Immunohistochemistry

The 5 μm human prostatic tissue sections were deparaffinized in xylene solution and rehydrated using gradient ethanol concentrations, and endogenous peroxidase activity was blocked with 3% hydrogen peroxide in methanol for 10 min. Heat-induced antigen retrieval was performed for all sections with 0.01-M sodium citrate/pH 6.0 at 98 °C for 30 min, and IHC staining with specific primary antibodies against α-SMA, collagen I, TGF-β1, CYP19, ERα, ERβ, GPER, HIF-1α, and Lox was performed.

### Masson’s trichrome staining

The 5 μm sections of prostatic tissues were deparaffinized in xylene (three washes for 3 min each) and hydrated in graded ethanol to distilled water. Slides were stained with a Masson’s trichrome-staining kit from ZSGB-BIO (Beijing, China), following the manufacturer’s protocol.

### Immunofluorescence

The samples, including human prostatic tissue sections, WPMY-1, and Prsc were grown on coverslips. The procedure for tissue immunofluorescence was the same as that for IHC. For cell immunofluorescence, 4% paraformaldehyde was used to fix cells for 15 min before incubating the cells with 0.5%Triton X-100 for 15 min. The remaining steps were the same as those outlined by IHC. The following primary antibodies were used (all diluted in blocking solution): 1:200 dilution mouse anti-α-SMA, and 1:100 dilution rabbit anti-collagen I. Anti-rabbit Alexa 488 and Anti-mouse Alexa 594 (ZSGB-BIO) secondary antibodies were used at 1:400 dilution. Samples were counterstained for 5 min with 1 mg/ml DAPI (ZSGB-BIO) in Tris-Buffered Saline/Tween 20. Fluorescent images were digitally captured using an Olympus IX71 photomicroscope at ×200 magnification.

### Quantitative PCR

Total RNA was extracted from WPMY-1 and Prsc using TRIzol (15596–018, Invitrogen). According to the manufacturer’s protocol, cDNA was synthesized from 1 µg RNA using a high-capacity cDNA reverse transcription kit (4368813, Applied Biosystems, Carlsbad, CA, USA). Quantitative real-time PCR (Q-PCR) was conducted using the GoTaq qPCR Master Mix (A6001, Promega, Madison, WI, USA) for a two-step cycling protocol with the Applied Biosystems 7500 Fast Real-Time PCR system to determine the mRNA expression levels of target genes. The relative expression levels were calculated using the 2 − ΔΔCt method. The quantities of all targets from the test samples were normalized to the GAPDH housekeeping gene.

### Western blot analysis

The expressions of specific genes were determined by the western blot. Briefly, cells were lysed in RIPA buffer, and proteins (20 µg) were separated on 8–10% SDS/PAGE gel and then transferred onto PVDF membranes (Millipore, Billerica, MA, USA). After blocking membranes with non-fat milk, they were incubated with appropriate dilutions (1:1000) of specific primary antibodies overnight at 4 °C; the blots were incubated with HRP-conjugated secondary antibodies and then visualized using the ECL system (ThermoFisher Scientific, Waltham, MA, USA).

### Cell proliferation assay

Cell viabilities were quantified on days 0, 2, 4, and 6 by incubating cells in 0.5 mg/ml of 3-(4,5-dimethylthiazol-2-yl)-2,5-diphenyltetrazolium bromide (MTT) (Sigma–Aldrich) for 2 h and dissolving in DMSO, followed by recording the absorbance at 570 nM.

### Lentivirus packaging

We used the vector Plvthm or PLKO plasmid for constructing lentivirus-transfected shRNA. The Plvthm-shGPER, Plvthm-HIF-1α, PLKO-shGαi1, PLKO-shGαi2, and PLKO-shGαi3 plasmids, and the PSPAX2-packaging plasmid and PMD2G envelope plasmid were transfected into 293 T cells using the standard calcium chloride transfection method for 48 or 72 h to obtain the lentivirus soup. The lentivirus soups were collected and concentrated by density gradient centrifugation and either used immediately or frozen at −80 °C for later use.

### Statistics

All statistical analyses were performed with SPSS 19.0 (SPSS Inc, Chicago, IL). The data values are presented as the mean ± SD of at least three independent experiments. Differences in mean values between the two groups were analyzed using a two-tailed Student’s *t*-test and the mean values of more than two groups were compared with one-way ANOVA. *P*-values < 0.05 were considered statistically significant.

## Supplementary information

Supplementary Figure Legends

Supplementary Figure 1

Supplementary Figure 2

Supplementary Figure 3

Original Data File

author checklist

## Data Availability

All data generated or analyzed during this study are included in this published article and its supplementary information files. The datasets used and/or analyzed during the current study are available from the corresponding author on reasonable request.
