# Supplementary material for: Estrogen and G protein-coupled estrogen receptor accelerate the progression of benign prostatic hyperplasia by inducing prostatic fibrosis
Source: Cell Death Dis. 2022 Jun 7;13(6):533. doi: 10.1038/s41419-022-04979-3 (PMC9174491; doi:10.1038/s41419-022-04979-3)
Supplement: Supplementary file 1 — Supplementary Figure Legends [file 41419_2022_4979_MOESM1_ESM.docx]

**Supplementary Figure 1.**

**(A)** IHC staining for TGF-β1 in the prostatic epithelium (up) and prostatic stroma (down) from the three groups. Scale bar: 50 μm. **(B)** WPMY-1 were treated with DHT at 0 nM(Ctr), 0.1 nM, 1 nM, and 10 nM for 72 h, and collagen I, α-SMA, and Lox were detected by western blot. GAPDH was used as a loading control.

**Supplementary Figure 2.**

**(A)** Microscope image of prostatic primary stromal cells. Scale bar: 50 μm. **(B)** Prostatic primary stromal cells were examined by immunofluorescence, cells were stained for Epcam (red), vimentin (green), and the nuclei were counterstained with DAPI (blue). Merged images indicate the fibroblast phenotype. Scale bar: 100 μm.

**Supplementary Figure 3.**

**(A)** The whole prostatic tissue slice stained with IHC for α-SMA and with Masson’s trichrome (serial section) and scanned by the APERIO AT2. **(B)** Flowchart demonstrating the calculation of the ratio of the stromal-to-epithelial area and the proportion of collagen. The images show only a part of the scanned prostatic tissue slice (the region indicated by black box in A).
