# Supplementary figures and images for: Estrogen and G protein-coupled estrogen receptor accelerate the progression of benign prostatic hyperplasia by inducing prostatic fibrosis
Source: Cell Death Dis. 2022 Jun 7;13(6):533. doi: 10.1038/s41419-022-04979-3 (PMC9174491; doi:10.1038/s41419-022-04979-3)

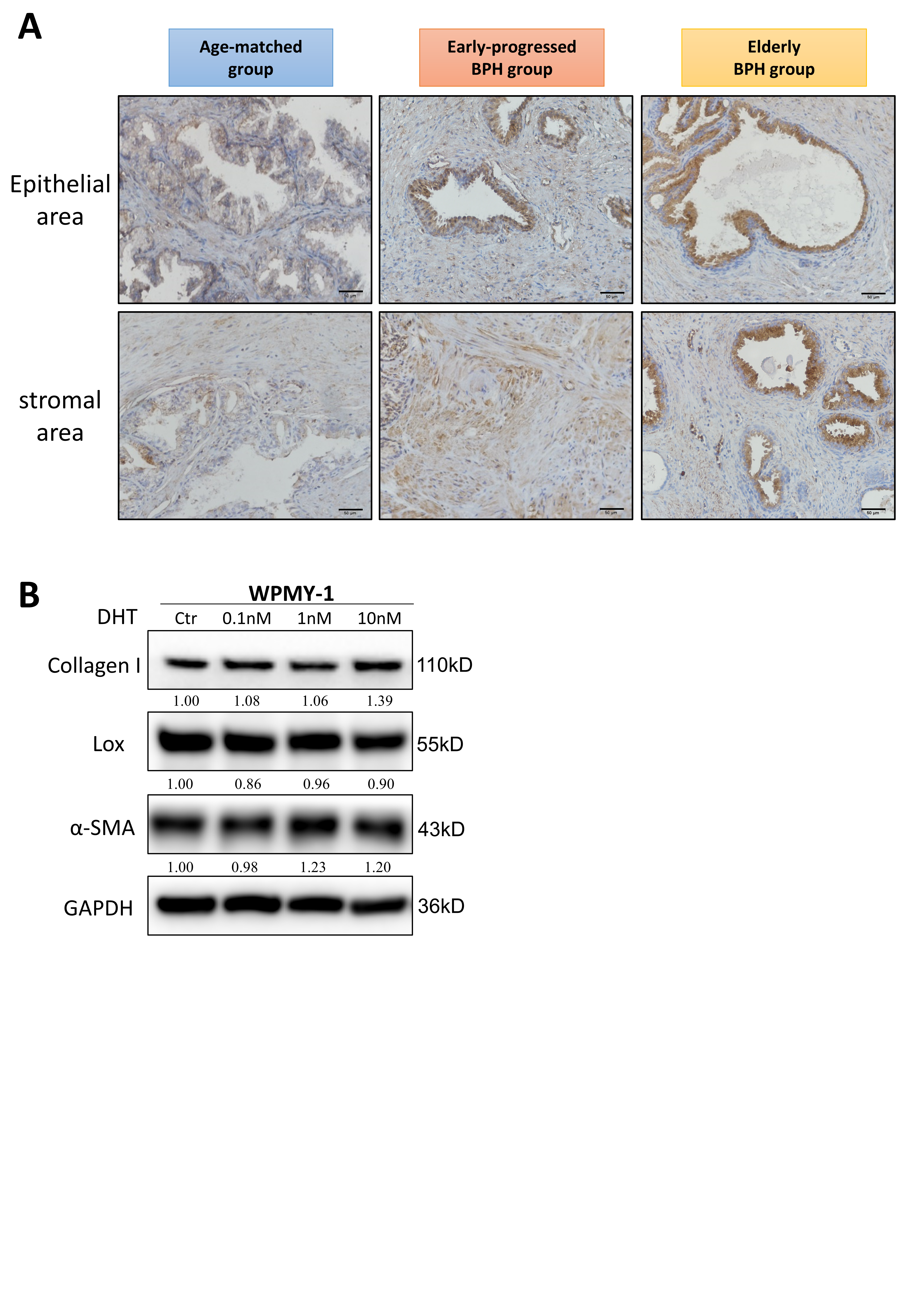

Supplement: Supplementary file 2 — Supplementary Figure 1 [file 41419_2022_4979_MOESM2_ESM.tif]

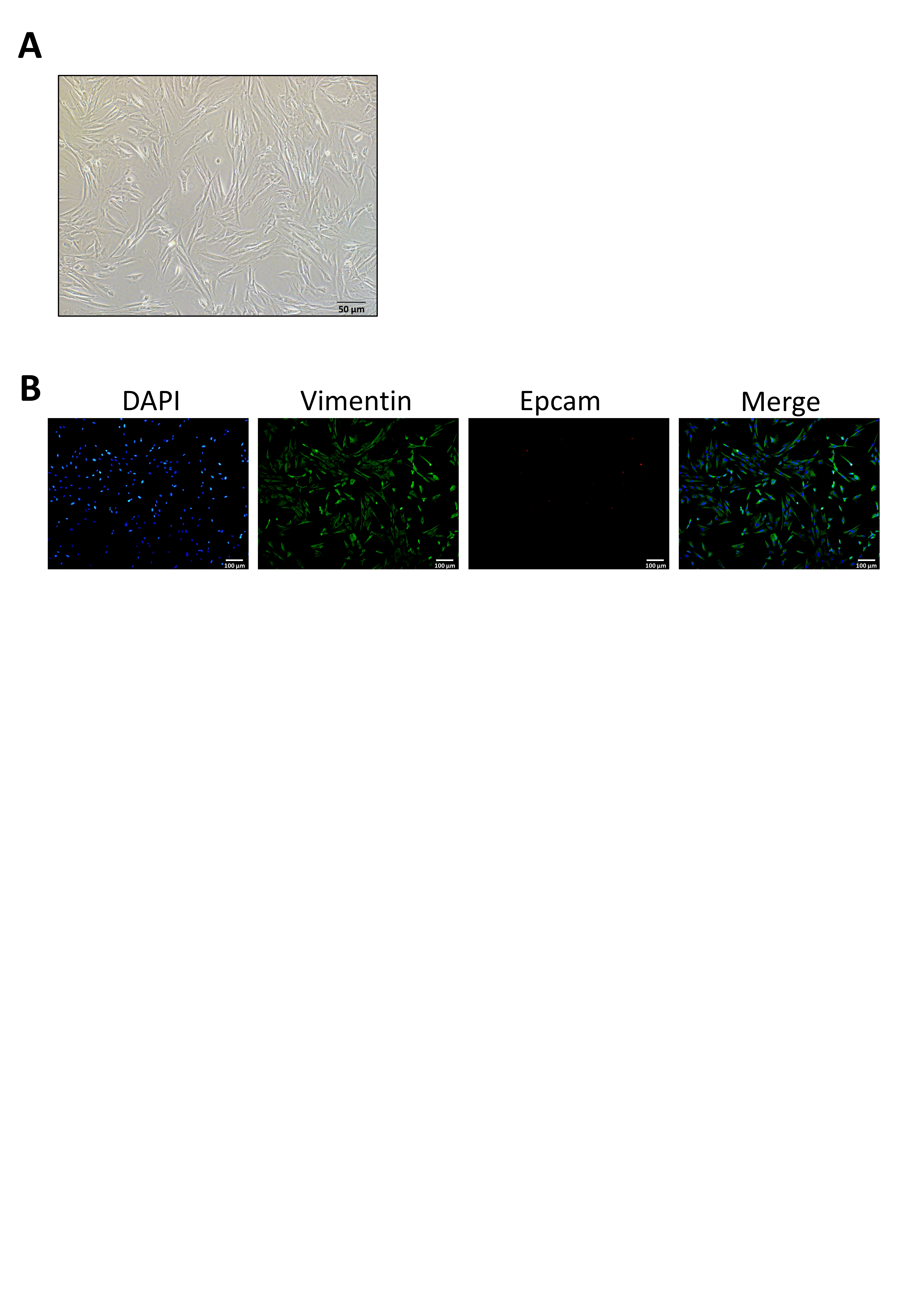

Supplement: Supplementary file 3 — Supplementary Figure 2 [file 41419_2022_4979_MOESM3_ESM.tif]

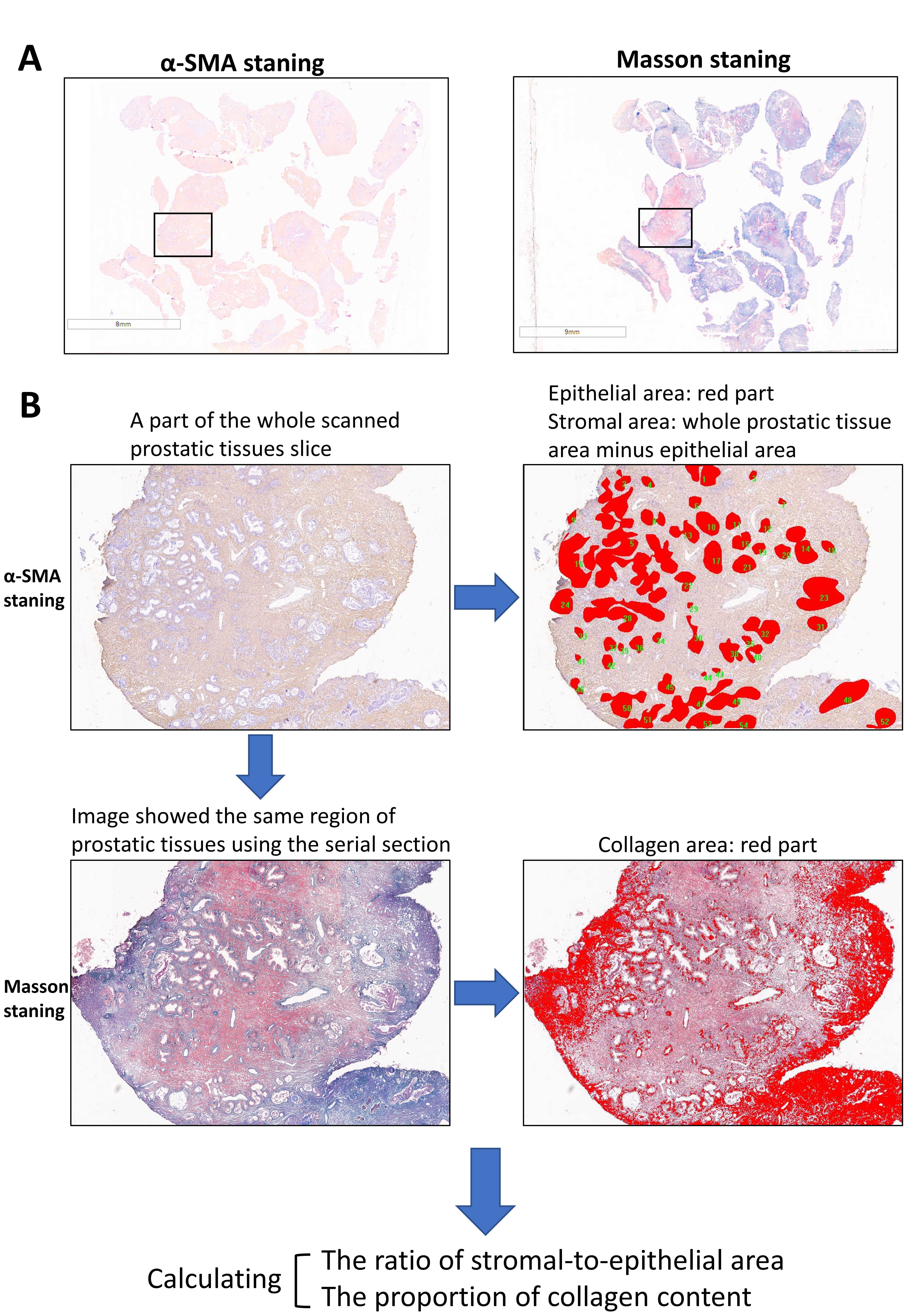

Supplement: Supplementary file 4 — Supplementary Figure 3 [file 41419_2022_4979_MOESM4_ESM.tif]
