# Supplementary material for: Estrogen and G protein-coupled estrogen receptor accelerate the progression of benign prostatic hyperplasia by inducing prostatic fibrosis
Source: Cell Death Dis. 2022 Jun 7;13(6):533. doi: 10.1038/s41419-022-04979-3 (PMC9174491; doi:10.1038/s41419-022-04979-3)

Fig. 3F

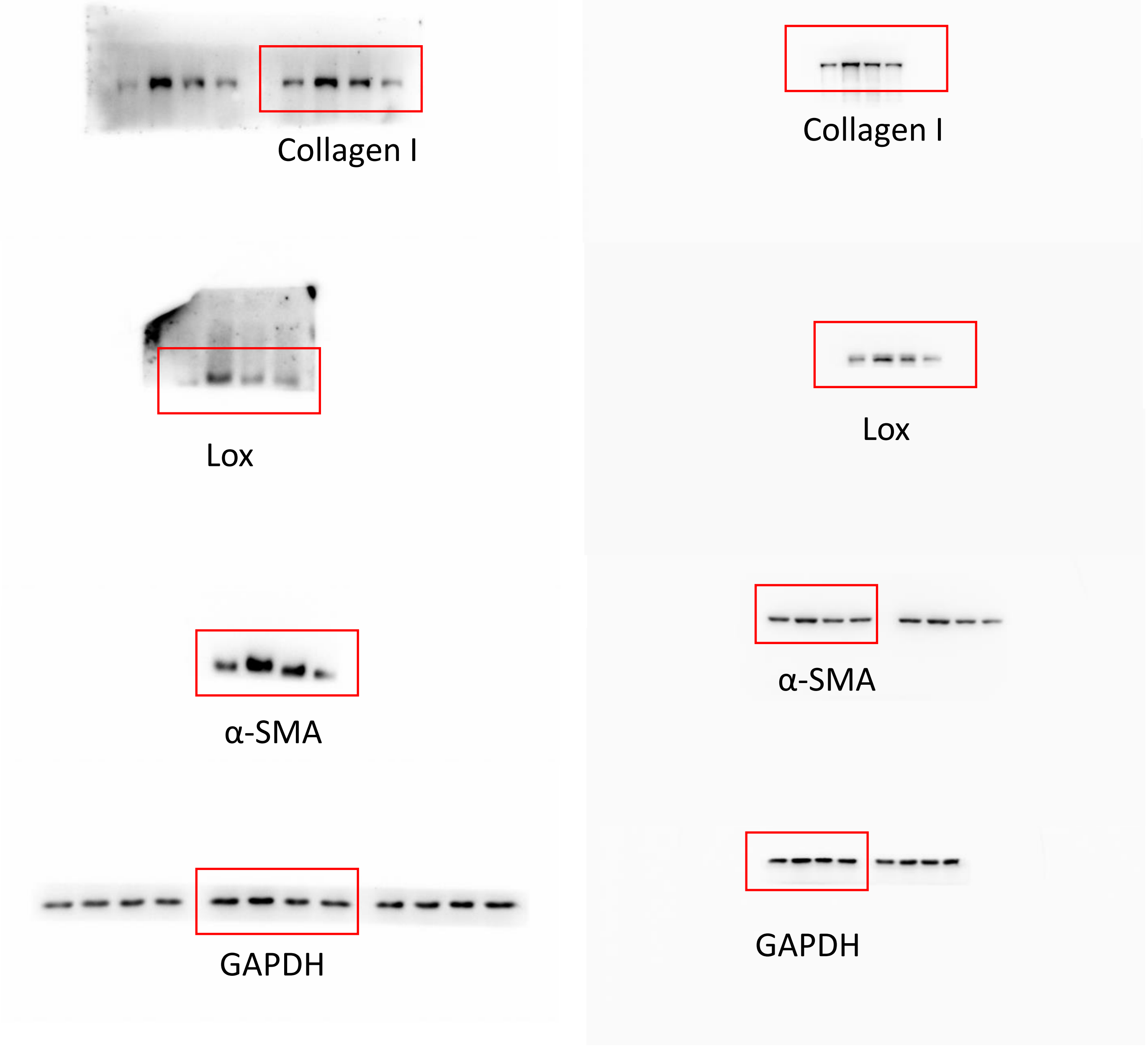

Fig. 3G

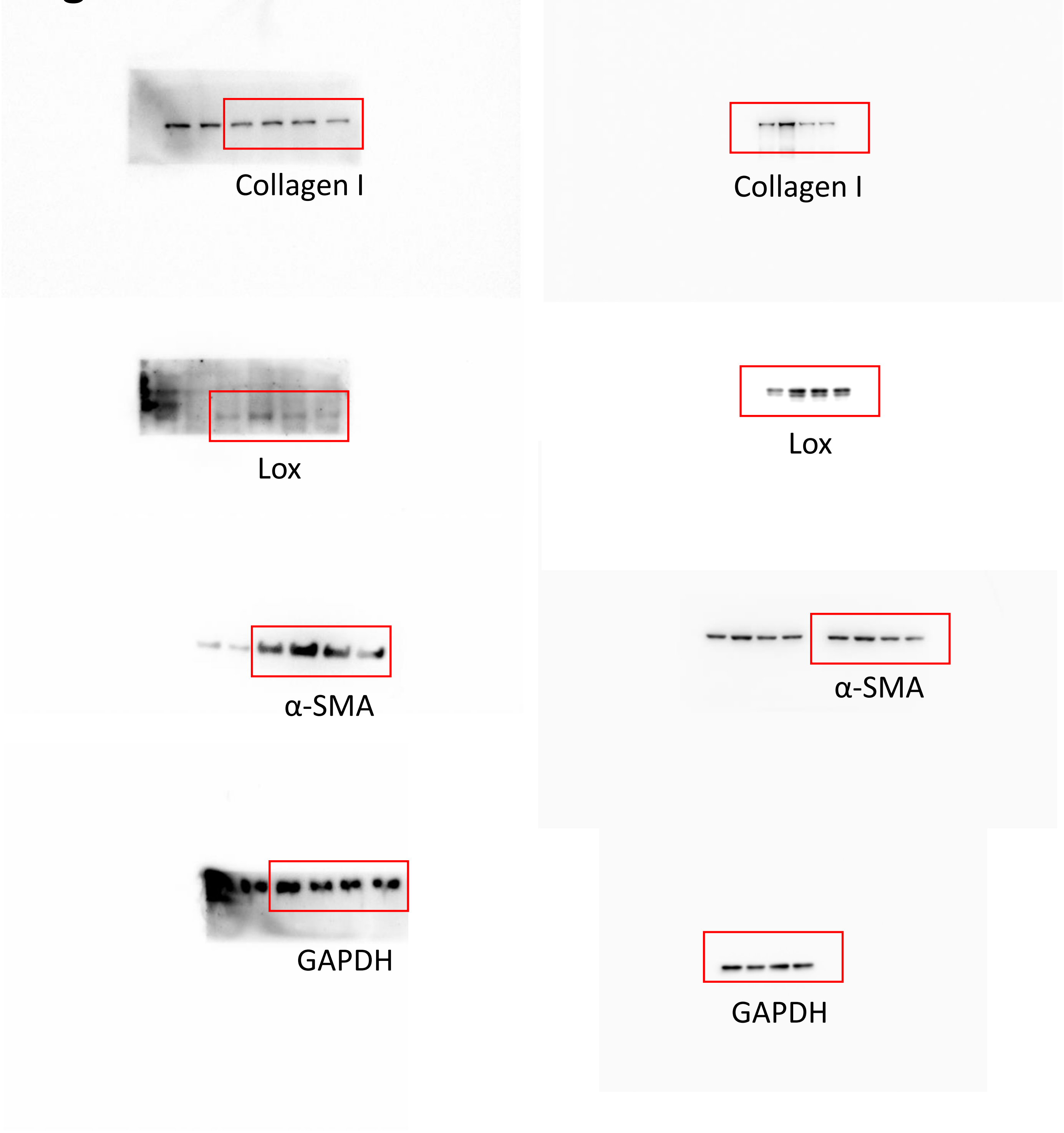

Fig. 3H

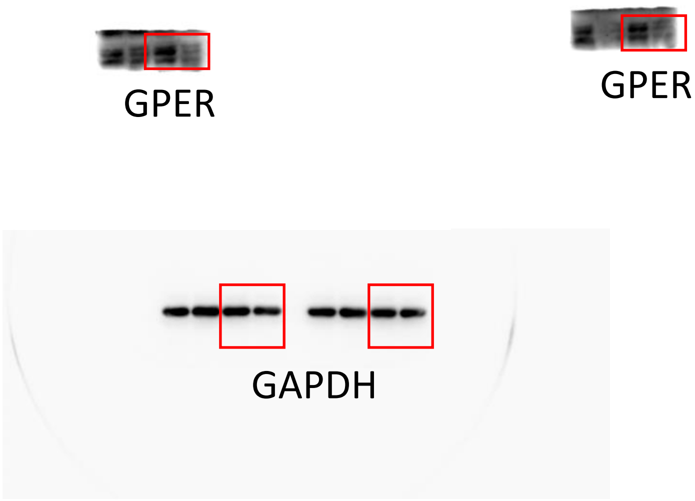

Fig. 3I

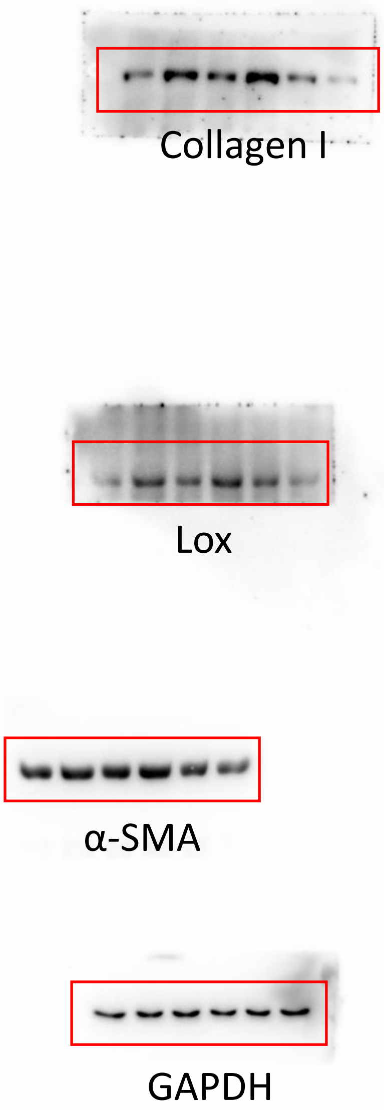

Fig. 3J

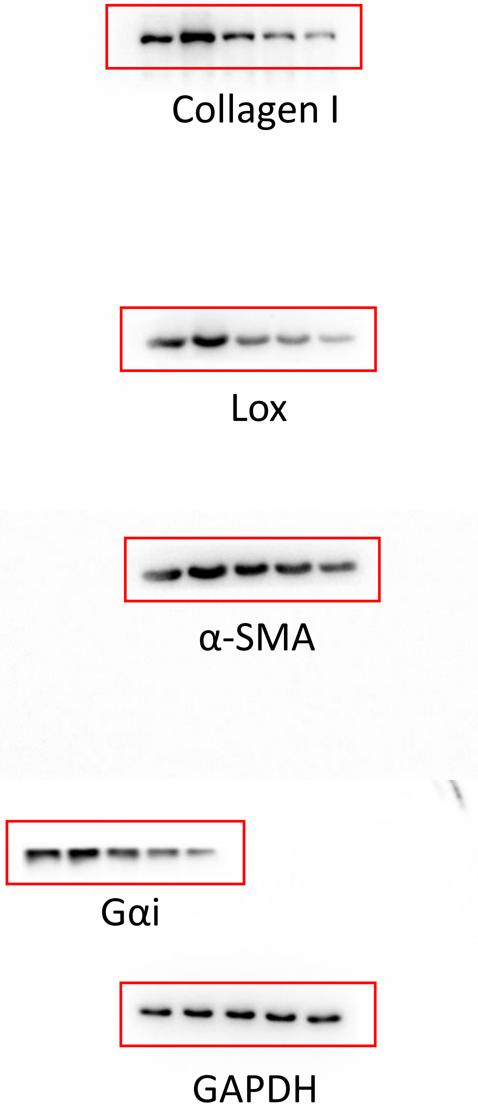

Fig. 4A

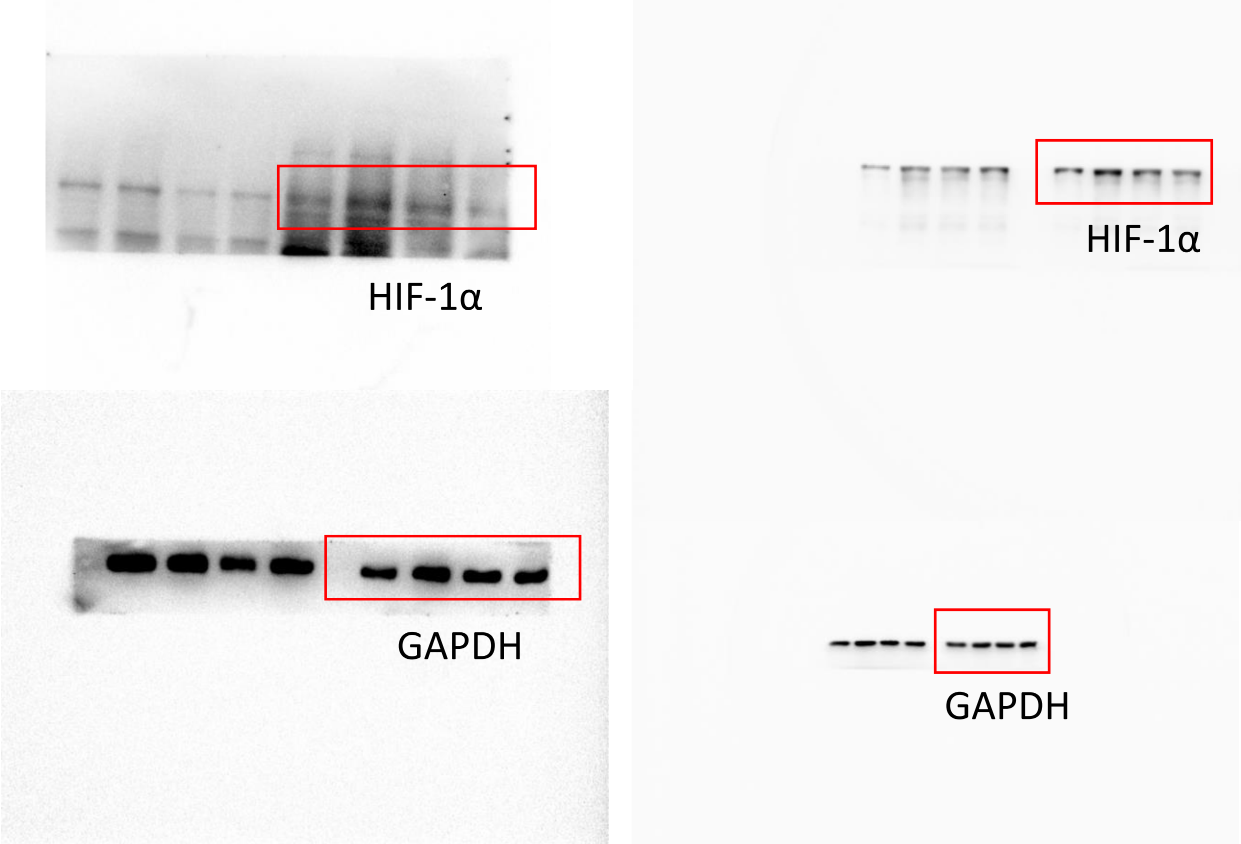

Fig. 4B

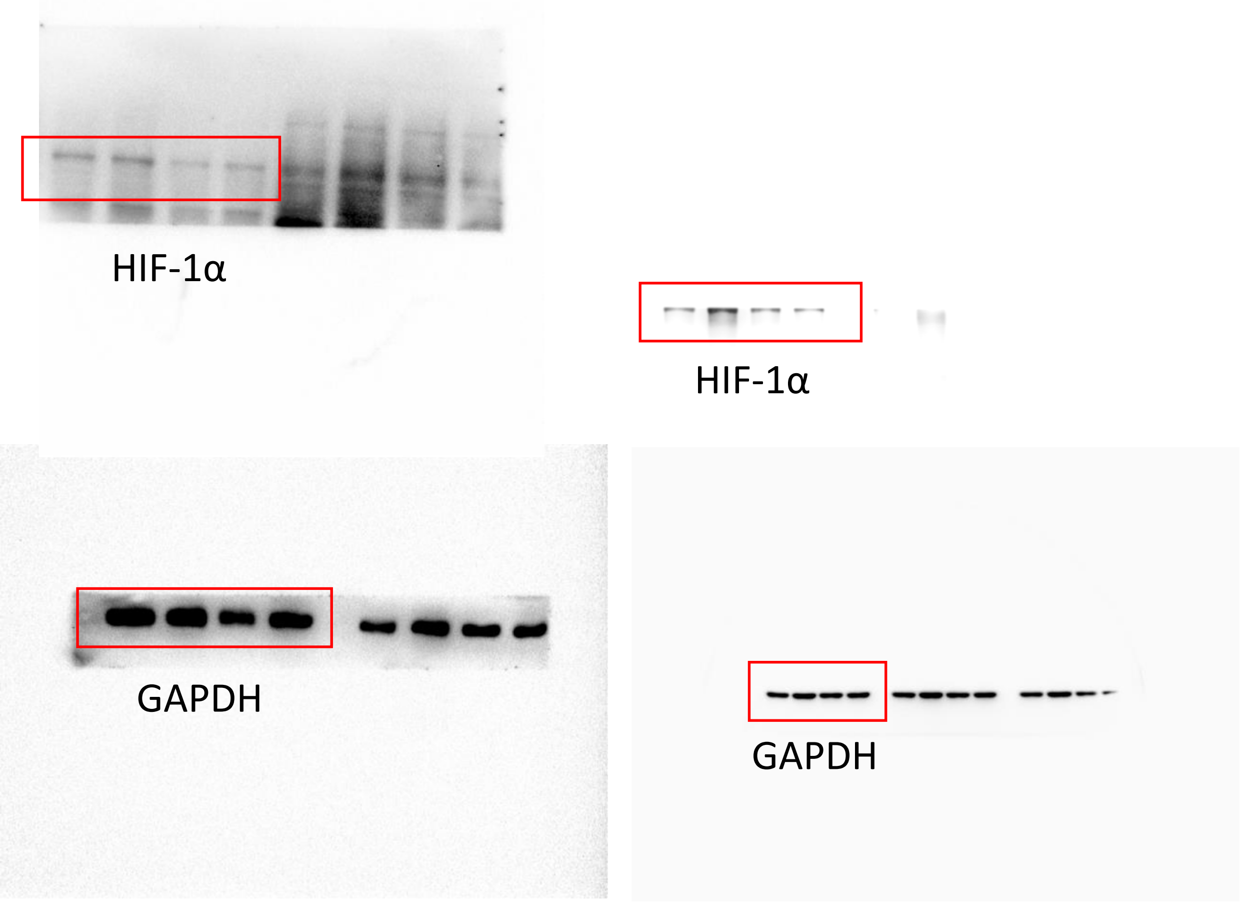

Fig. 4C

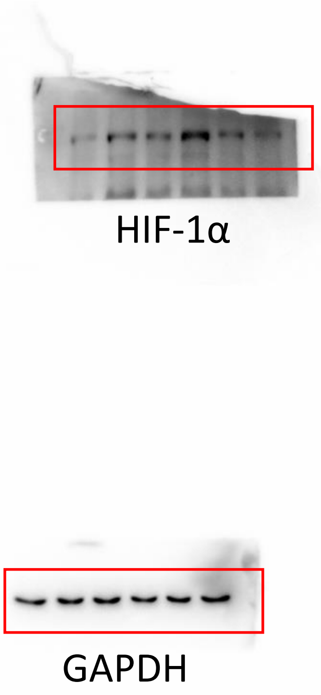

Fig. 4D

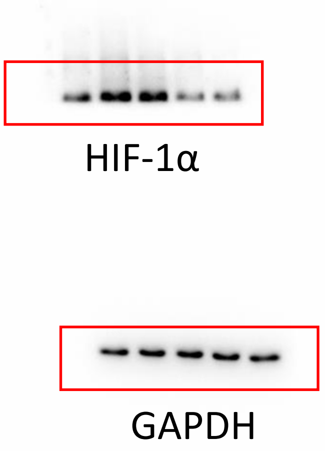

Fig. 4F

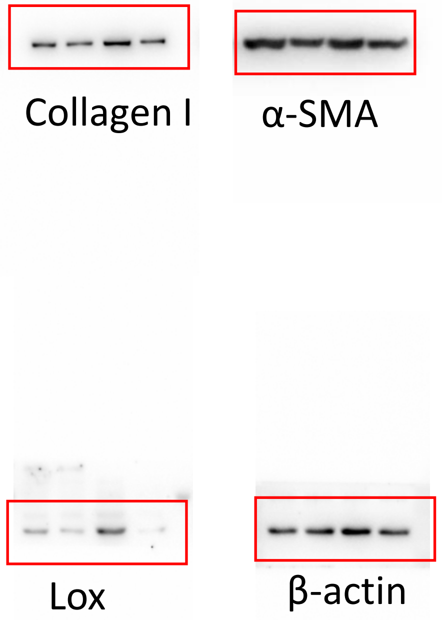

Fig. 4H

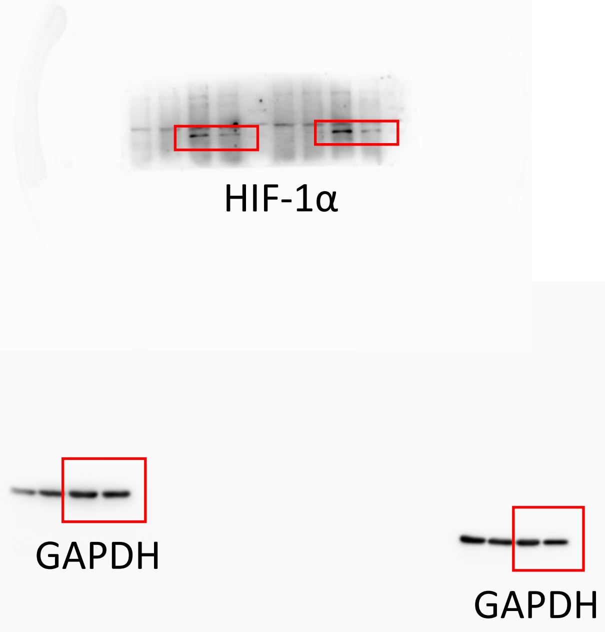

Fig. 4I

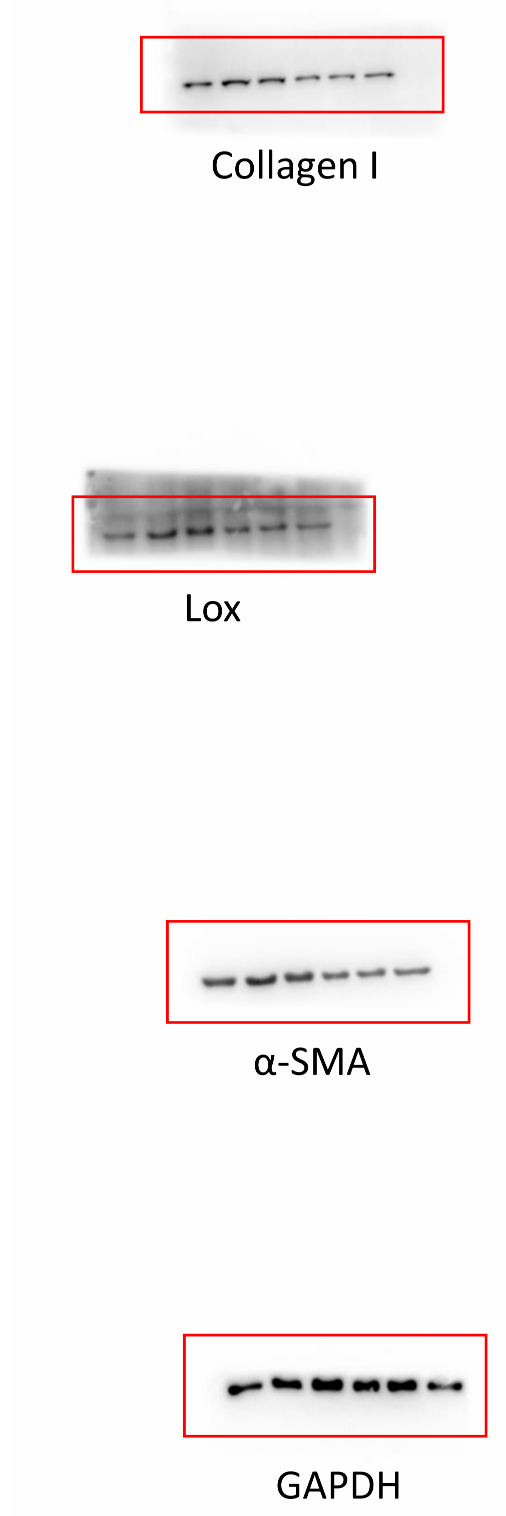

Fig. 4J

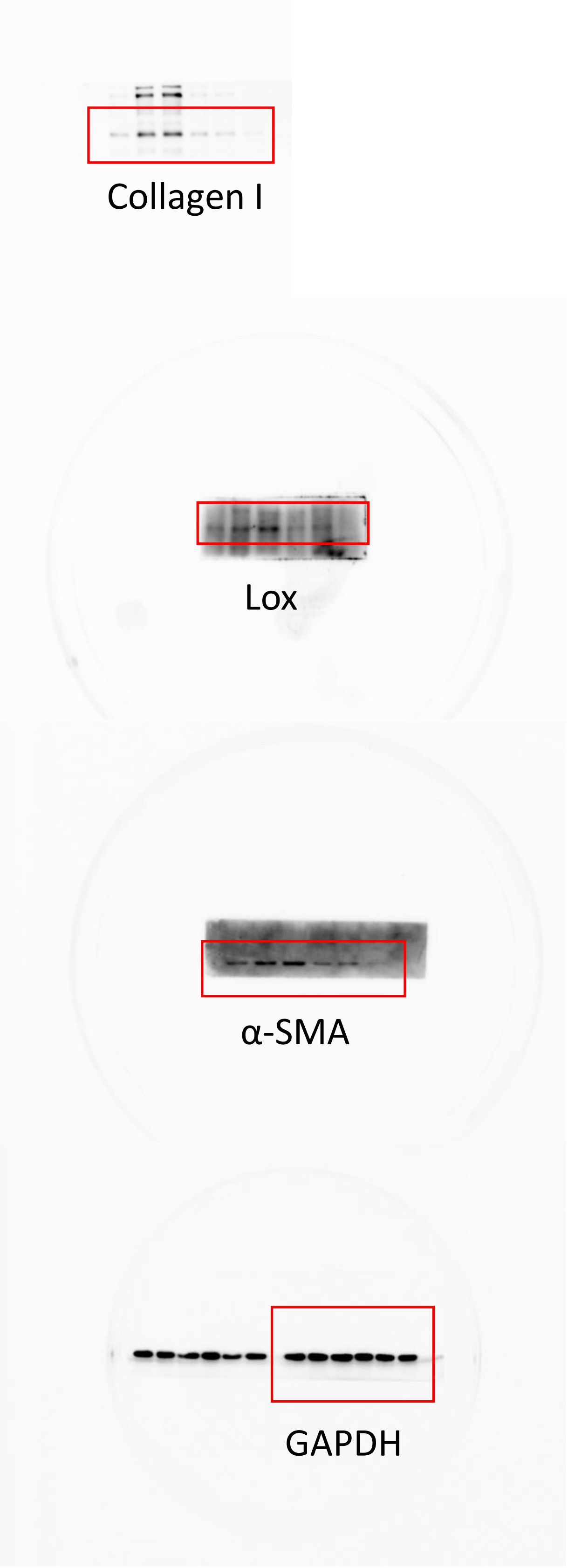

Fig. 5A

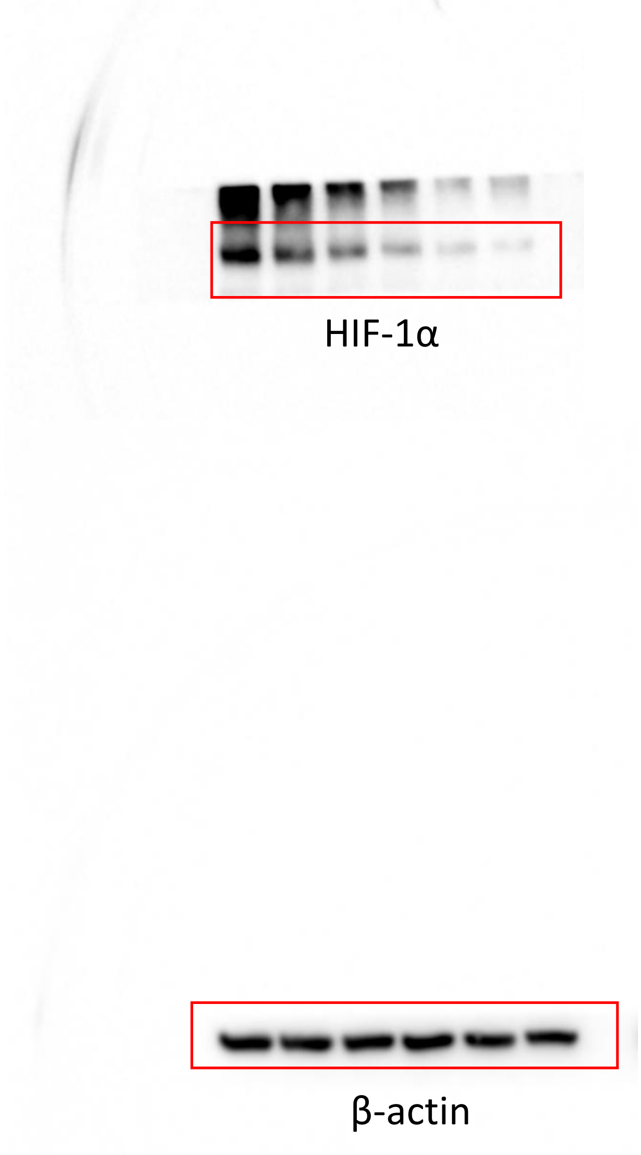

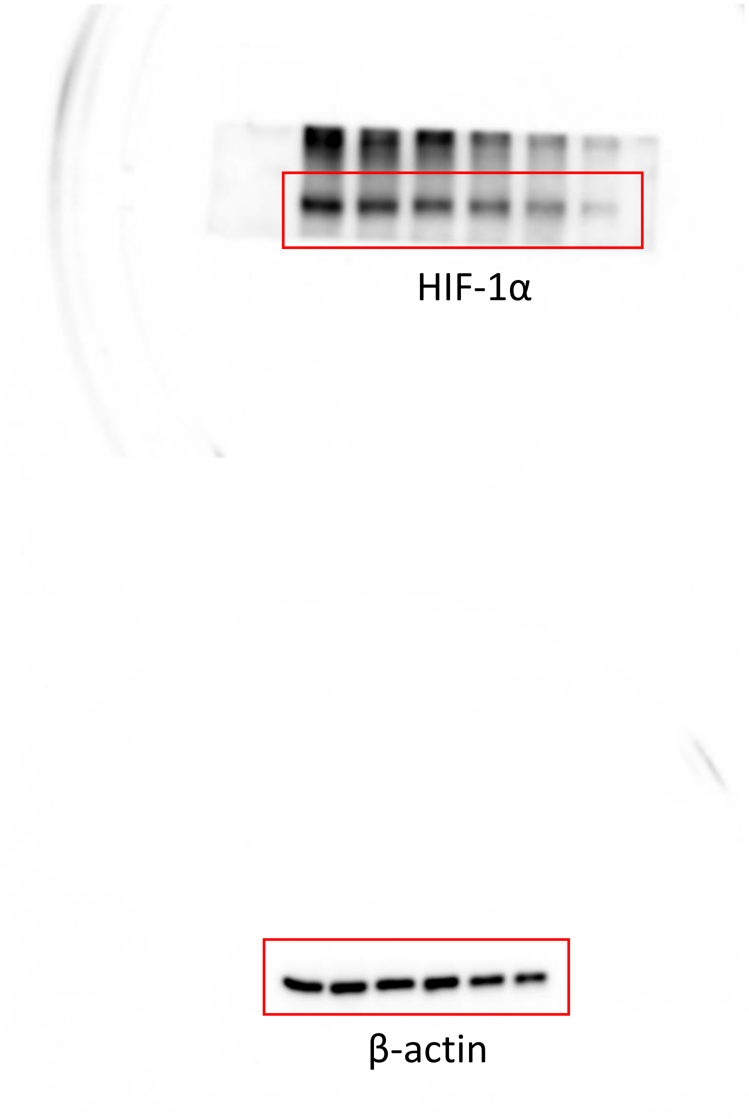

Fig. 5B

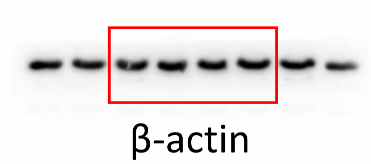

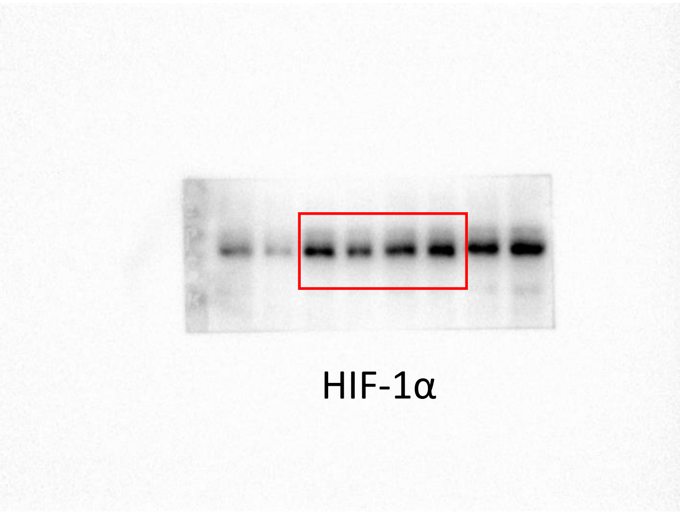

Fig. 5C

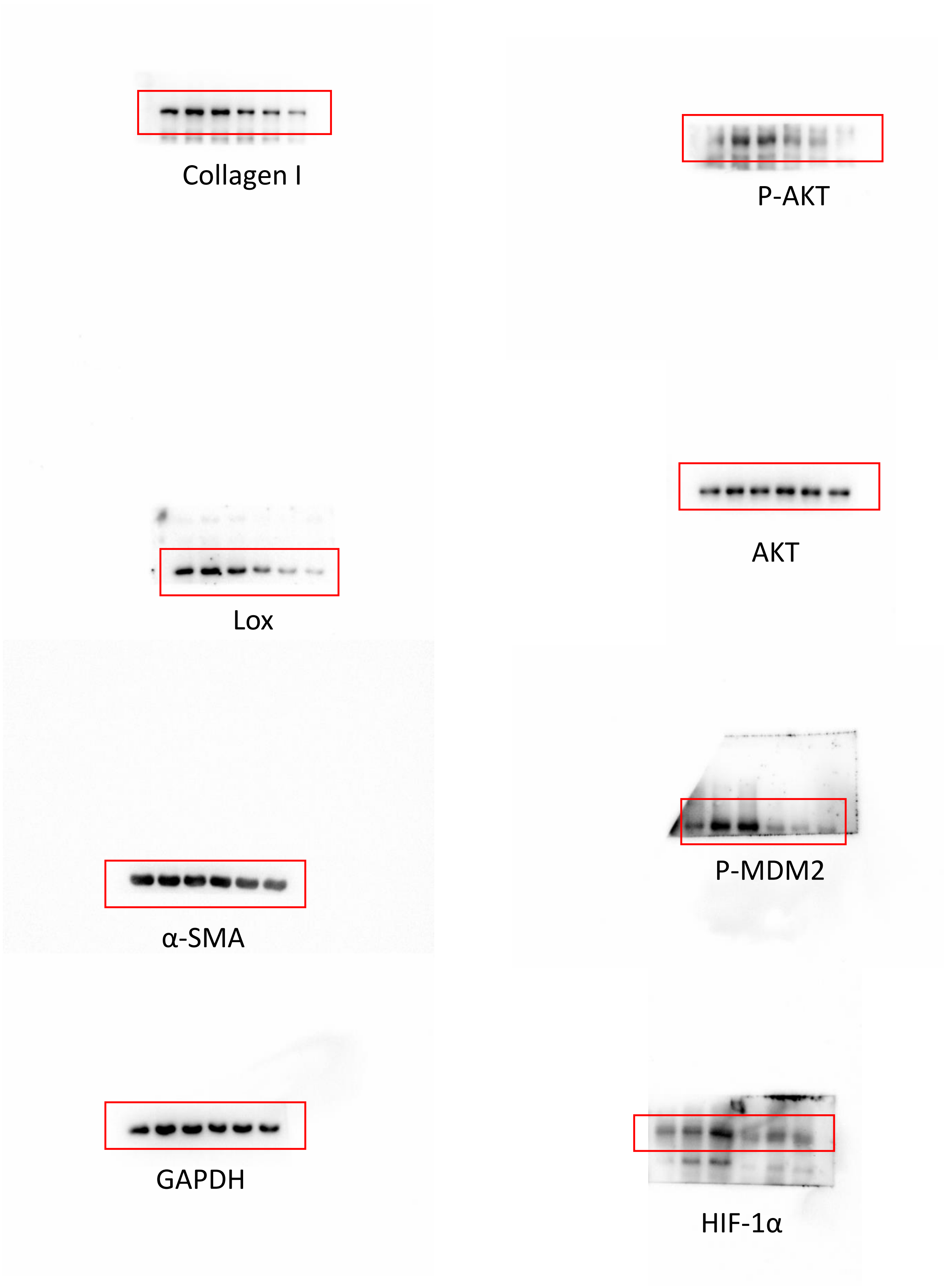

Fig. 6E

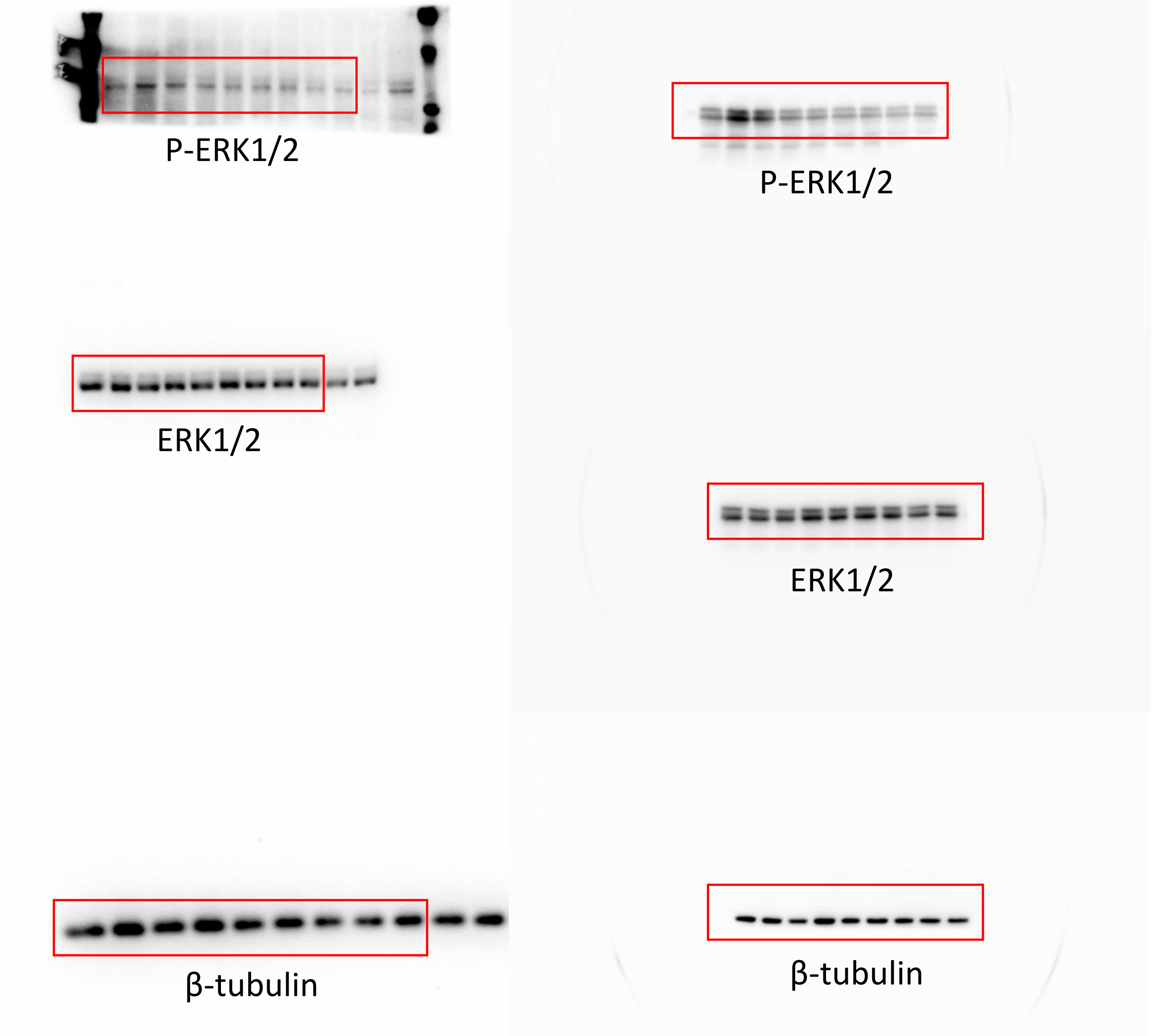
P-ERK1/2
P-ERK1/2
ERK1/2
ERK1/2
β-tubulin
β-tubulin

Fig. 6F

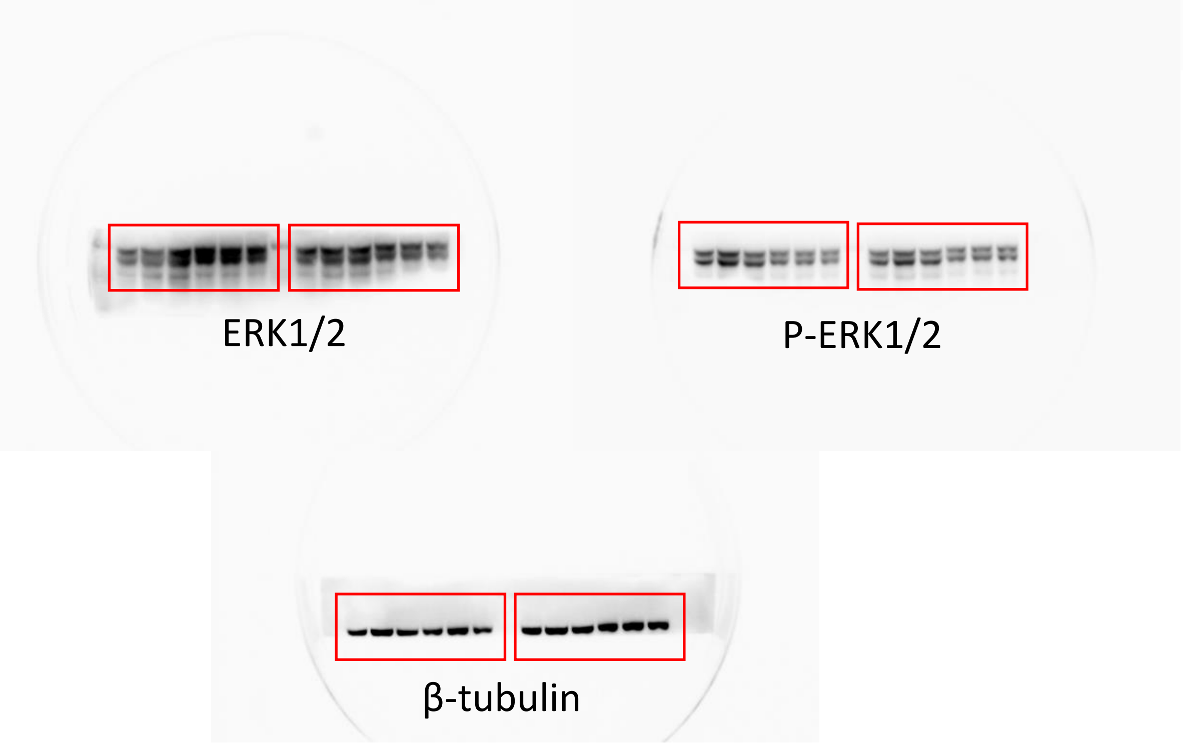
ERK1/2
P-ERK1/2
β-tubulin

Fig. 6G

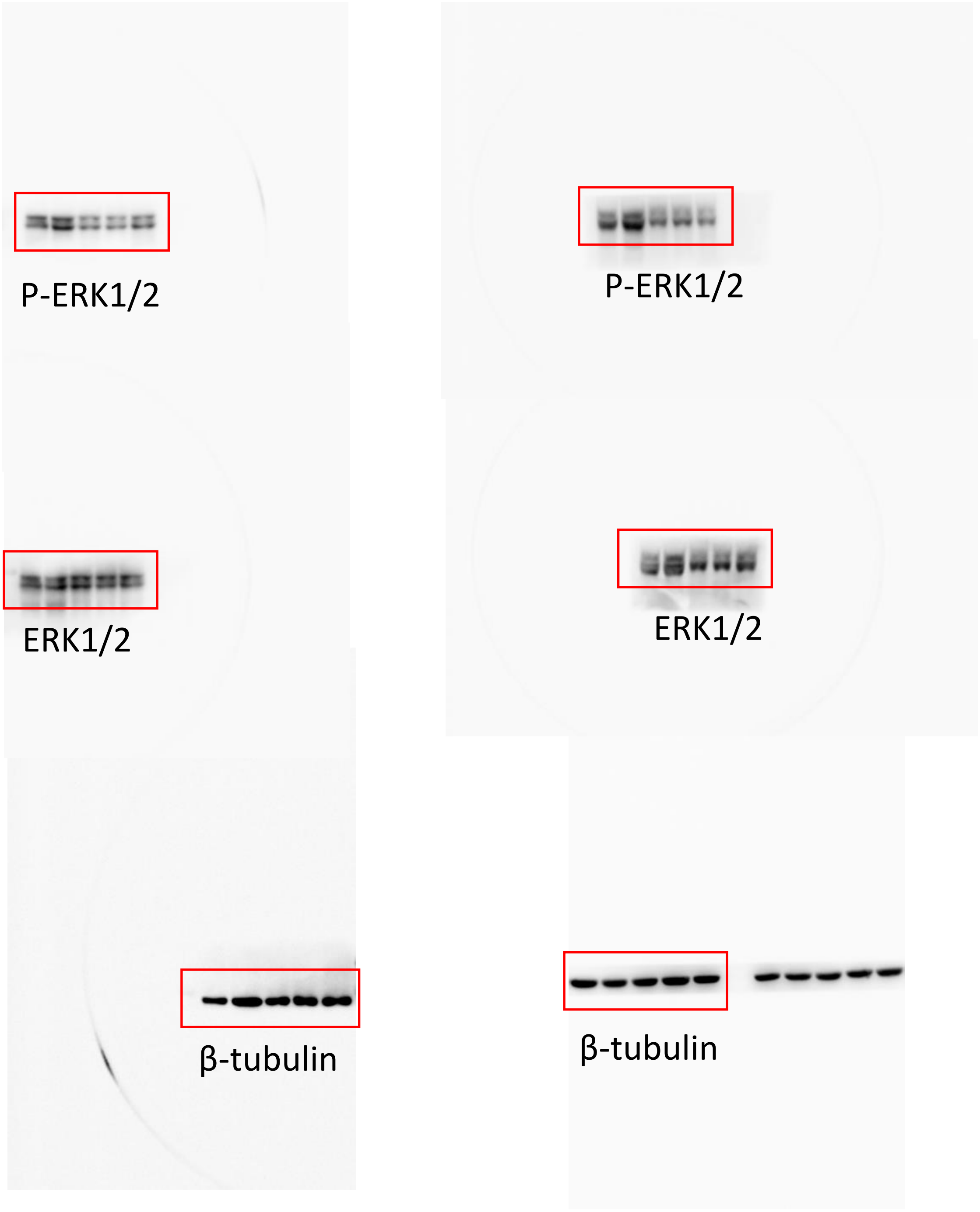

Supplementary Figure. 1B

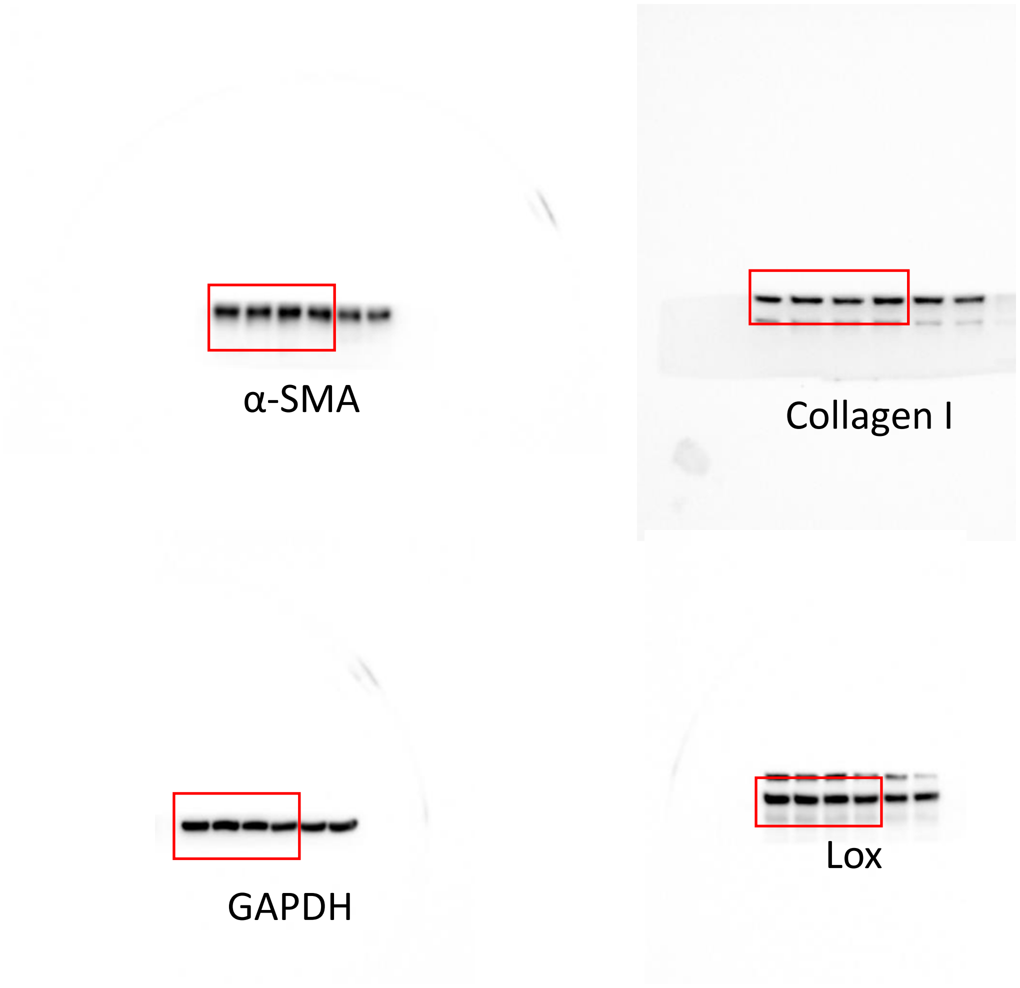

Supplement: Supplementary file 5 — Original Data File [file 41419_2022_4979_MOESM5_ESM.pdf]
